# Transcriptional inhibition of STAT1 functions in the nucleus alleviates Th1 and Th17 cell-mediated inflammatory diseases

**DOI:** 10.3389/fimmu.2022.1054472

**Published:** 2022-12-15

**Authors:** Jiyoon Park, Min-Ji Son, Chun-Chang Ho, Su-Hyeon Lee, Yuna Kim, Jaekyeung An, Sang-Kyou Lee

**Affiliations:** ^1^ Department of Biotechnology, Yonsei University of Life Science and Biotechnology, Seoul, South Korea; ^2^ Good T Cells, Inc., Seoul, South Korea

**Keywords:** signal transducer and activator of transcription 1 (STAT1), Th1 cells, Th17 cells, psoriasis, inflammatory bowel disease (IBD), transcription modulation, autoimmune disease (AD)

## Abstract

T helper 1 cells (Th1 cells) and T helper 17 cells (Th17 cells) play pivotal roles in the pathogenesis of various autoimmune diseases, including psoriasis and inflammatory bowel disease (IBD). Signal transducer and activator of transcription 1 (STAT1) regulates the Th1 and Th17 cell lineage commitment at an early stage and maintains their immunological functions *in vitro* and *in vivo*. The previous strategies to block STAT1 functions to treat autoimmune diseases inhibit Th1 cell activity but simultaneously cause hyper-activation of Th17 cells. Herein, to modulate the functions of pathogenic Th1 and Th17 cells without genetic modification in normal physiological conditions, we generated the nucleus-deliverable form of the transcription modulation domain of STAT1 (ndSTAT1-TMD), which can be transduced into the nucleus of the target cells in a dose- and time-dependent manner without affecting the cell viability and T cell activation signaling events. ndSTAT1-TMD significantly blocked the differentiation of naïve CD4^+^ T cells into Th1 or Th17 cells *via* competitive inhibition of endogenous STAT1-mediated transcription, which did not influence Th2 and Treg cell differentiation. When the gene expression profile of Th1 or Th17 cells after ndSTAT1-TMD treatment was analyzed by mRNA sequencing, the expression of the genes involved in the differentiation capacity and the immunological functions of Th1 or Th17 cells were substantially reduced. The therapeutic potential of ndSTAT1-TMD was tested in the animal model of psoriasis and colitis, whose pathogenesis is mainly contributed by Th1 or/and Th17 cells. The symptoms and progression of psoriasis and colitis were significantly alleviated by ndSTAT1-TMD treatment, comparable to anti-IL-17A antibody treatment. In conclusion, our study demonstrates that ndSTAT1-TMD can be a new therapeutic reagent for Th1/17 cell-mediated autoimmune diseases by modulating the functions of pathogenic Th1 and Th17 cells together.

## Introduction

CD4^+^ T helper (Th) cells play an essential role in various immune responses and inflammation. The homeostatic imbalance of the functional immune network caused by hyper-activated effector CD4^+^ T cells has been implicated in the onset and maintenance of the inflammatory responses that lead to severe autoimmune diseases. More specifically, interferon-γ (IFN-γ)-secreting T helper 1 (Th1) cells and interleukin-17 (IL-17)-secreting T helper 17 (Th17) cells are known to be associated with the pathogenesis of organ-specific inflammatory diseases, including psoriasis and inflammatory bowel diseases (1, 2).

Th1 cells are known to protect our body against invaded intracellular pathogens and viruses by secreting IFN-γ, in turn activating both macrophages and dendritic cells (DCs). The secreted IFN-γ also acts as an inducer of Th1 differentiation by activating the signaling pathway of STAT1 at an early stage (3). Th17 cells mainly secrete IL-17 and IL-23, which are involved in the clearance of extracellular bacteria and fungi. Moreover, the existence of IFN-γ during Th17 cell lineage development dampens the full commitment of Th17 cell differentiation (4). Aside from all these properties that Th1 and Th17 cells distinctively possess, both overactivated Th1 and Th17 cells play critical roles in autoimmune disorders by disrupting the immunological balance between helper T cells and regulatory T cells, accompanied by subsequent cytokine dysregulation (5, 6).

STAT1, a member of the STAT family, regulates the axes of Th1/Th2 and Th1/17 cell differentiation by controlling an expression pattern of the T helper subset-specific genes during an early stage of CD4^+^ T cell differentiation (7, 8). The STAT1 consists of six domains, including an N-terminal domain (NTD), a coiled-coil domain (CCD), DNA binding domain (DBD), a linker domain (LD), SCR2 homology domain (SH2), a transactivation domain (TAD). The intracellular signaling by the binding of IFN-γ and IL-27 to their respective receptors on the surface of naïve CD4^+^ T cells triggers STAT1 to be activated and homodimerized (9, 10). The STAT1 homodimer is translocated to the nucleus to induce and maintain the T-bet expression, a transcription factor involved in Th1 responses (11). STAT1 has also been shown to suppress Th17 cell differentiation by directly binding to the *Rorc* or *Il17a* loci, which are the corresponding chromosomal gene location of each ROR-γt and IL-17A expression critically involved in the regulation of Th17 responses (8). STAT1-knockout (KO) studies have reported that the loss of endogenous STAT1 impairs the Th17 cell lineage commitment (12). Previously, functional inhibition of STAT1 using genetic methods in various studies has been continuously suggested as a therapeutic strategy for Th1 or Th17 cell-mediated systemic autoimmune disorders and showed the substantial effect of restricting Th1 cells. However, no significant functional modulation of Th17 cells was detected, and Th17 cells in the absence or low level of STAT1 exhibited the hyper-activated phenotype (13).

Psoriasis is a relapsing and inflammatory skin disorder characterized by a thick and non-pruritic plaque covered by a silvery scale on the skin (14). Inflammatory bowel disease (IBD), including Chron’s disease (CD) and ulcerative disease (UC), is characterized by dysregulated intestinal immune responses against gut microbiota followed by inflammation in the gastrointestinal (GI) tract, with typical symptoms of persistent diarrhea, rectal bleeding, or weight loss (15, 16). The exact mechanism of how psoriasis and IBD develop remains unclear. However, hyper-activated Th1 and Th17 cells are estimated as the leading cause of irregular immune responses in the skin or intestinal tract (16, 17). In addition, recent studies revealed that IFN-γ and IL-17A, secreted from Th1 and Th17 cells, respectively, are crucial mediators to induce and accelerate the pathological progression of psoriasis and IBD (18–20). Yet, the therapeutic strategies of targeting IFN-γ or IL-17A for psoriasis and IBD still face several limitations to overcome.

Here, we generated the nucleus-deliverable form of the transcription modulation domain of STAT1 (ndSTAT1-TMD), which is the fusion protein between TMD of STAT1 and human-origin protein transduction domain (PTD), Hph-1 (21–23). ndSTAT1-TMD can be delivered into the nucleus of the cells efficiently without cytotoxicity and affecting TcR-mediated signaling events. ndSTAT1-TMD specifically inhibited the transcriptional activity of endogenous STAT1 in Th1 and Th17 cells and showed a therapeutic potential comparable to that of anti-IL-17A antibody when administered to psoriasis and IBD mouse models. Our studies suggest that ndSTAT1-TMD can be a new and efficacious therapeutics reagent for Th1/17 cell-mediated autoimmune diseases.

## Methods and materials

### DNA cloning for the generation of recombinant proteins

The plasmid of mouse STAT1 (BC004808) was purchased from Origene Technologies, Inc, USA. The sequence encoding amino acids from 317 to 488 of the wildtype STAT1 DBD were amplified by PCR and ligated into pET28a (+) expression vector, which contains Hph-1-PTD, 6x His tag, and FLAG tag. The point-mutated form of DBD (V426D, T427D) was generated through pfu DNA polymerase (Agilent). All cloned DNA constructs generated were sequenced in order to confirm the fidelity of the open reading frame following cloning.

### Expression and Purification of STAT1-TMD, ndSTAT1-TMD (V426D, T427D), and ndSTAT1-TMD

All recombinant proteins were expressed in the BL21 Codon Plus (DE3) RIPL strain (Invitrogen, Waltham, MA, USA) of *Escherichia coli* and purified through affinity chromatography. Cells expressing STAT1-TMD, ndSTAT1-TMD, or ndSTAT1-TMD (V426D, T427D) were collected in native lysis buffer (10 mM imidazole, 50 mM NaH2PO4, 300 mM NaCl, pH 8.0) and sonicated. Inclusion bodies were pelleted *via* centrifugation of cell lysates and were further sonicated in native lysis buffer containing 3 M Urea (10 mM imidazole, 50 mM NaH2PO4, 300 mM NaCl, 3 M Urea, pH 8.0). Cell lysates were pelleted, and the supernatants were mixed with Ni-NTA resin (Qiagen). The recombinant proteins were washed with buffer containing 30 mM imidazole, 50 mM NaH2PO4, 300 mM NaCl, pH 8.0, and eluted with buffer containing 500 mM imidazole, 50 mM NaH2PO4, 300 mM NaCl, pH 8.0. The eluted recombinant proteins were further purified to remove endotoxin *via* ion exchange chromatography and desalted with PD-10 Sephadex G-25 columns (GE Healthcare). The purified recombinant proteins contained safe levels of endotoxins at approximately 6 EU/ml. During subsequent animal experiments, no animals showed signs of immune responses against endotoxins, such as anaphylactic shock.

### Western blot

Purified recombinant proteins were separated through sodium dodecyl sulfate-polyacrylamide gel electrophoresis (SDS-PAGE) and transferred onto polyvinylidene fluoride (PVDF, Bio-Rad, Hercules, CA, USA) membrane. The membrane was blocked with 5% Bovine Serum Albumin in 0.1% Tween-20 in Tris-buffered saline. The recombinant proteins were incubated with anti-6x His-tag antibody (27E8 Clone, Cell Signaling, Danvers, MA, USA) and anti-mouse IgG-HRP antibody (Abcam, Cambridge, MA, USA). ECL reagent (Bio-Rad) was used to visualize the proteins in ChemiDoc (Bio-Rad).

### Cell culture

HEK293T cells were cultured in DMEM medium (Lonza, Basel, Switzerland) supplemented with 10% heat-inactivated fetal bovine serum (FBS, Hyclone), 2 mM L-glutamate (Lonza), 100 μg/ml penicillin-streptomycin (Lonza), 1 mM sodium pyruvate (Lonza), and NEAA (Gibco, Waltham, MA, USA).

All primary cells were maintained in RPMI 1640 (Lonza) medium supplemented with 7.5% heat-activated fetal bovine serum (FBS, Hyclone), 2 mM L-glutamate (Lonza), 100 μg/ml penicillin-streptomycin (Lonza), and 50 μM β-Mercaptoethanol (Sigma Aldrich, St. Louis, MO, USA). All cells were cultured at 37°C in a humidified atmosphere of 5% CO_2_.

### Protein treatment

2x10^5^ mouse splenocytes were cultured with the recombinant protein on a 96-well cell culture plate (Eppendorf) in a dose-dependent manner (0.5-2 μM, 1 hr) or in a time-dependent manner (2 μM, 0-72 hr) and used in flow cytometry. 2x10^6^ mouse splenocytes were seeded on a coverslip placed on the bottom of a 12-well cell culture plate (SPL, Pocheon, South Korea) and incubated with 1 μM of ndSTAT1-TMD or STAT1-TMD for an hour before analysis and observed using a confocal microscope (LSM 980, Carl Zeiss, Jena, Germany). 2x10^5^ mouse naïve CD4^+^ T cells were cultured on a 96-well cell culture plate (Eppendorf) under appropriate T cell subset-polarizing conditions with the addition of recombinant proteins in a dose-dependent manner (0.5-2 μM).

### Immunocytochemistry

2x10^6^ mouse splenocytes were seeded on a round microscope cover glass 18 mm in diameter, which was placed on the bottom of a 12-well cell culture plate (SPL). Cells were cultured with media containing PBS, 1 μM of STAT1-TMD, or 1 μM of ndSTAT1-TMD in a 12-well cell culture plate (SPL) for an hour. After recombinant protein treatment, cells were washed with PBS, fixed with 10% formalin solution (Sigma-Aldrich), and permeabilized with 0.5% Triton X-100 solution (Sigma-Aldrich). The samples were blocked with 1% BSA solution. The delivered proteins were captured with anti-FLAG (DYKDDDDK) Alexa 488 antibody (Invitrogen). After washing with PBS, the nuclei were counterstained with 4’,6-diamino-2-phenylindole (DAPI), and cells were visualized with a confocal microscope (LSM 980, Carl Zeiss).

### Cell cytotoxicity assay

Cell viability following ndSTAT1-TMD and ndSTAT1-TMD (V426D, T427D) treatment was tested using Cell Counting Kit-8 (CCK-8; Dojindo Laboratories, Kumamoto, Japan). 2x10^5^ mouse splenocytes were seeded into a 96-well culture plate (SPL) and incubated with varying doses of ndSTAT1-TMD or ndSTAT1-TMD (V426D, T427D) for an hour. After protein treatment, CCK-8 reagent was added to the cultured cells and incubated for an additional 4 hr. Cell viability was measured using a microplate reader (Bio-Rad) at an absorbance of 450 nm wavelength.

### CD4^+^ T cell differentiation

CD4^+^ CD62L^+^ T Cell Isolation Kit (Miltenyi Biotec, Bergisch Gladbach, Germany) was used to purify CD4^+^ CD62L^+^ naïve T cells in RBC-eliminated splenocytes from the spleen of 8-week-old female C57BL/6N (B6) mice. Isolated CD4^+^ naïve T cells were activated in a 96-well cell culture plate (Eppendorf) coated with 1 μg/ml of anti-CD3ϵ (BD Bioscience, San Diego, CA, USA) and anti-CD28 (BD Bioscience) for 72 hr. To differentiate CD4^+^ naïve T cells into Th1, Th2, Th17, or Treg cells, different cytokine mixtures, and antibodies were added to the culture plates: Th1 (25 ng/ml of recombinant mouse IL-12 and 2 μg/ml of anti-IL-4 antibody), Th2 (200 ng/ml of recombinant mouse IL-4 and 2 μg/ml of anti-IFN-γ antibody), Th17 (1 ng/ml of recombinant mouse TGF-β1, 25 ng/ml of recombinant mouse IL-6, 2 μg/ml of anti-IFN-γ antibody, and 2 μg/ml of anti-IL-4 antibody), and iTreg (5 ng/ml of recombinant mouse TGF-β and 20 ng/ml of recombinant mouse IL-2). Cytokines and antibodies were obtained from PeproTech (Rocky Hill, NJ, USA), BioLegend (San Diego, CA, USA), and R&D Systems (Minneapolis, MN, USA). All cells were incubated at 37°C in a humidified atmosphere of 5% CO_2_.

### ELISA

The supernatants of activated or differentiated CD4^+^ effector T cells were collected to measure the level of cytokines. IFN-γ, IL-4, IL-17A, and IL-2 levels were measured by ELISA following the manufacturer’s protocol (Invitrogen).

Mouse serum samples were obtained through centrifuging blood samples acquired from IMQ-induced psoriasis mice on day 7 of disease induction or dextran sulfate sodium (DSS)-induced colitis mice on day 12 of disease induction. TNF-α, IL-1β, and IL-6 levels in blood were measured by ELISA following the manufacturer’s protocol (Invitrogen).

The concentration of cytokines was measured using a microplate reader (Bio-Rad) at an absorbance of 450 nm wavelength.

### Reporter gene analysis

ndSTAT1-TMD and ndSTAT1-TMD (V426D, T427D) were tested for the binding ability to the target gene promoter. 5x10^5^ HEK293T cells were seeded into a 6-well culture plate (SPL) and co-transfected with 1 μg of pCMV6 vector containing mouse wildtype STAT1 and 1 μg of IL-17A promoter-luciferase vector using Lipofectamine Reagents (Invitrogen) diluted in Opti-MEM (Gibco). After 4 hr incubation at 37°C in a 5% CO_2_, cells were treated with ndSTAT1-TMD or ndSTAT1-TMD (V426D, T427D) in a dose-dependent manner (0.5-2 μM). Cells were then incubated overnight, washed with PBS, and lysed using Cell Culture Lysis 5X Reagent (Promega, Madison, WI, USA). Cell lysates were mixed with Luciferase Assay Substrate (Promega), and luciferase activity was measured by a luminometer (Promega).

### mRNA-sequencing and gene set enrichment analysis

Mouse CD4^+^ T cells differentiated under Th1- or Th17-polarizing conditions with or without the addition of ndSTAT1-TMD were harvested after 72 hr. Macrogen (Seoul, South Korea) performed RNA extraction and sequencing. Trimmomatic 0.38 program were used to trim out low-quality data, adaptor, and DNA contamination of the first readout. Then, using the HISAT2 program, trimmed data were mapped out to the genomic reference (10mm). The transcript assembly was performed using the reference-based aligned reads information, and transcripts per million (TPM) counts were also acquired from assembled transcripts through StringTie.

Gene set enrichment analysis (GSEA) was performed on the ndSTAT1-TMD-treated sample groups using GSEA v4.1.0. MSigDB was used as a gene sets database, including Hallmark gene sets (24) and ImmuneSigDB (25, 26). The number of permutations was set to 1000 and the permutation type to ‘gene set’. The chip platform was set to ‘Mouse_Gene_Symbol_Remapping_Human_Orthologs_MSigDB.v7.4. chip’. TPM values for 17358 genes from 4 samples of ndSTAT1-TMD-treated and non-treated Th1 cells and 17201 genes from 4 samples of ndSTAT1-TMD-treated and non-treated Th17 cells were used for the analysis.

### Animals

8-9 weeks-old female and male C57BL/6N (B6) mice (8-9 weeks) and 9-week-old female C57BL/6J (B6) mice were purchased from Orient Bio (South Korea). All mice were housed and maintained in the semi-specific-pathogen-free (SPF) facility of Yonsei Laboratory Animal Research Center (YLARC). All animal studies were approved by the Institutional Animal Care and Use Committee (IACUC) of Yonsei Laboratory Animal Research Center (YLARC) and conducted under the guidelines of YLARC-IACUC for ethical usage. (IACUC-A-202109-1327-01, IACUC-A-202201-1404-01).

### Psoriasis induction and scoring of psoriasis area and severity index

Following a week of acclimatization, 8-week-old female C57BL/6J mice were shaved and completely unhaired with depilation cream on the back with a 2*4 cm area on day 1. The normal group applied Vaseline on exposed back skin (40 mg) and both ear skin (20 mg/ear) for 7 consecutive days. And the disease-induction groups were applied with 5% IMQ cream (Aldara cream, Dong-A S&T, Inc. Seoul, South Korea) on exposed back skin (40 mg) and both ears skin (20 mg/ear) from day 1 to day 7. ndSTAT1-TMD (20 or 60 μg/mouse), ndSTAT1-TMD (V426D, T427D) (60 μg/mouse), or anti-IL-17A antibody (BioXCell, Lebanon, NH, USA) (60 μg/mouse) were administrated by intraperitoneal injection every other day from day 1 to day 5, and the body weight and the psoriasis area and severity index (PASI) score were measured daily from day 1 to day 7. The exposed back skin of mice was photographed on day 5 and day 7, and both ears of mice were photographed on day 7. The thickness of the back or right ear skin was measured by Vernier caliper for 7 consecutive days. The psoriasis area and severity index (PASI) score of all groups was evaluated daily and measured in a blinded fashion according to the following index (27). Erythema (Redness): 0, fleshy pink; 1, minor reddening across the surface; 2, medium red across the surface with dark red patches; 3, dark red across the surface with dark red patches. Induration (Thickness): 0, 1-5%; 1, 5-50%; 2, 50-100%; 3, >100%. Desquamation (Scaling): 0, no skin flaking; 1, minor dry spots without flaking; 2, dry spots across a majority of the skin with flaking; 3, dry spots across a majority of the skin with moderate flaking across a large surface area and severe flaking.

The protocol of the experiments was accepted by the Institutional Animal Care and Use Committee (IACUC) of Yonsei University (South Korea), and the experiments for the psoriasis mouse model were performed with the IACUC guidelines for the ethical use of animals (IACUC-A-202201-1404-01).

### Colitis Induction and Scoring of disease activity index

Following a week of acclimatization, 8-week-old male C57BL/6N mice were given either sterile water or water containing 2.5% DSS from day 1 to day 6. The DSS-containing water was changed every other day. After day 6, all mice were given sterile water. Mice were intraperitoneally injected with ndSTAT1-TMD (20 or 100 μg/mouse), ndSTAT1-TMD (V426D, T427D) (100 μg/mouse), or anti-IL-17A antibody (BioXCell, Lebanon, NH, USA) (100 μg/mouse) once daily from day 6 to day 11, and the body weight and disease activity index (DAI) scores were measured daily from day 1 to day 11. All mice were sacrificed for analysis on day 12. The colon (from the cecum to the rectum) was isolated to measure the length. The following index was used to give blind measurements of DAI scores: Body weight change: 0, 0-1%; 1, 1-5%; 2, 5-10%; 3, 10-20%; 4, >20%. Stool: 0, normal; 1, some soft but still formed; 2, very soft; 3, diarrhea. Fecal occult blood: 0, normal; 1, positive hemoccult; 2, blood traces in stool; 3, rectal bleeding.

The protocol of the experiments was accepted by the Institutional Animal Care and Use Committee (IACUC) of Yonsei University (South Korea), and colitis experiments were followed by the IACUC guidelines for the ethical use of animals (IACUC-A-202109-1327-01).

### Histological examination

In the IMQ-induced psoriasis mice model, the 1 cm^2^ of the back skin and the right ear were obtained from each mouse and fixed in 4% phosphate-buffered paraformaldehyde, embedded in paraffin, and stained with hematoxylin and eosin (H&E) and Masson’s trichrome. The histological samples were imaged through an optical microscope (Olympus CX40) (Olympus Corporation, Shinjuku, Tokyo, Japan). The epidermal thickness of the back skin or right ear skin was measured by Image J program.

In the DSS-induced colitis mice model, the distal section of the colon was obtained from each mouse, fixed with 4% phosphate-buffered paraformaldehyde. Each sample was then embedded in paraffin, sectioned horizontally or vertically, and stained with H&E and periodic acid-Schiff (PAS). The histological samples were imaged through an optical microscope (Olympus BX51) (Olympus Corporation). The following index was used to give blind measurements of the degree of inflammatory cell infiltration, crypt damage, and goblet cell depletion. Inflammatory cell infiltration: 0, no infiltration; 1, mild infiltration <25%; 2, moderated infiltration <50%; 3, marked infiltration >50%. Crypt damage: 0, none; 1, some crypt damages; 2, larger spaces between crypts; 3, large areas without crypts. Goblet cell depletion: 0, none; 1, mild depletions <25%; 2, moderate depletions <50%; 3, marked depletions >50%. The muscle layer thickness of the colon was measured by Image J program.

All tissue staining was performed in Korea CFC (Seoul, South Korea).

### Flow cytometry

The transduction efficiency of ndSTAT1-TMD in mouse splenocytes was analyzed through intracellular staining with anti-FLAG-PE (BioLegend) after protein treatment for the indicated length of time.

For analysis, activated CD4^+^ T cells were stained with either anti-CD69-FITC (BD Bioscience) or anti-CD25-PE (BD Bioscience). Following 72 hr incubation with the recombinant proteins, the differentiated CD4^+^ effector T cell subsets were restimulated with Cell-Stimulation Cocktail (500X) (eBioscience, San Diego, CA, USA) for 4 hr. Each subset was then fixed and permeabilized with Fixation/Permeabilization Buffer (eBioscience) and stained intracellularly for appropriate lineage-defining transcription factors and cytokines. All antibodies were purchased from eBioscience.

Isolated immune cells from the spleen or draining lymph node (dLN) of IMQ-induced psoriasis mice and the spleen or mesenteric lymph node (mLN) of DSS-induced colitis mice were restimulated with Cell Stimulation Cocktail (500X) (eBioscience) for 4 hr. Following restimulation, cells were first stained with anti-CD4-AF700 (eBioscience) before fixation and permeabilization. Intracellular cytokines and transcription factors were further stained with anti-IFN-γ-APC, anti-IL-17A-APC, anti-T-bet-PE, anti-ROR-γt-PE, or anti-Foxp3-PE (eBioscience).

Skin-infiltrating leukocytes from the back and ear skin of IMQ-induced psoriasis mice were isolated. Their properties were analyzed by staining appropriate surface markers or staining intracellular transcription factors or cytokines. For intracellular staining, cells were restimulated using Cell Stimulation Cocktail (500X) (eBioscience) for 4 hr and fixed and permeabilized. The cells were stained with anti-CD45-AF700, anti-CD11b-APC, anti-IFN-γ-PE, anti-IL-17A-PE, anti-T-bet-PE, anti- ROR-γt-PE (eBioscience), and anti-F4/80-PE (BD Bioscience).

All cells restimulated using Cell Stimulation Cocktail (500x) were incubated at 37°C in a humidified atmosphere of 5% CO_2_. Flow cytometry was performed using SA3800 (Sony, Tokyo, Japan), and the Flowjo V10 program was used to analyze the data.

### Nitro oxide production assay

The blood was obtained from IMQ-induced psoriasis mice of each group on day 7 from disease induction, and the serum was separated by centrifugation. The nitro oxide (NO) production level was measured using Griess Reagent System and according to the manufacturer’s protocol (Peprotech). The nitro oxide (NO) level of blood supernatant samples was measured by analyzing the absorbance at 540 nm using a microplate reader (Bio-Rad).

### Isolation of skin-infiltrating leukocytes

Skin-infiltrating leukocytes were isolated from the back skin or left ear skin of IMQ-induces psoriasis mice (28). After 7 days of IMQ application, the 1 cm^2^ area from the mouse back skin and left ear were cut off, removed with remaining IMQ cream by HBSS (Gibco) wash and separated with epidermis and dermis using curved forceps. Separated epidermis and dermis were digested with Dispase II solution (5 mg/ml, Sigma Aldrich) at 37°C. After first digestion, the dermis was cut quickly into small pieces (< 0.5 mm) with curved scissors in Dermis Dissociation buffer (DMEM containing 1 mg/ml of collagenase P and 100 μg/ml of DNaseI (Hyclone, Roche) and incubated at 37°C for 1 hr. The fully digested dermal suspension was filtered through a 40-μm cell strainer (Falcon) and rinsed with DMEM medium containing 10% FBS (Hyclone). Isolated dermal-infiltrating leukocytes were collected by centrifugation. The epidermis, after first digestion, was cut quickly into small pieces (< 2 mm) with curved scissors and transferred to the 0.05% TE (Trypsin-EDTA, Hyclone) buffer and incubated at 37°C for 5 min. After incubation, trypsin neutralizing solution (5% FBS diluted with HBSS) was added to the fully digested epidermis, and the epidermal suspension was filtered through a 40-μm cell strainer (Falcon). Isolated epidermal-infiltrating leukocytes were collected by centrifugation.

### Statistical analysis

The results are expressed as a mean ± SEM (n≥3). An unpaired Student’s t-test or ANOVA analysis was used for statistical analysis of group differences, followed by Dunnett’s multiple comparison test. The number of asterisks demonstrated the following significance: ns; not significant, *p<0.05, **p<0.01, ***p<0.001, and ****p<0.0001. GraphPad Prism 9 was used for the analysis of all data.

## Results

### Intracellular delivery kinetics of ndSTAT1-TMD into the nucleus of primary T cells

**Figure 1:**
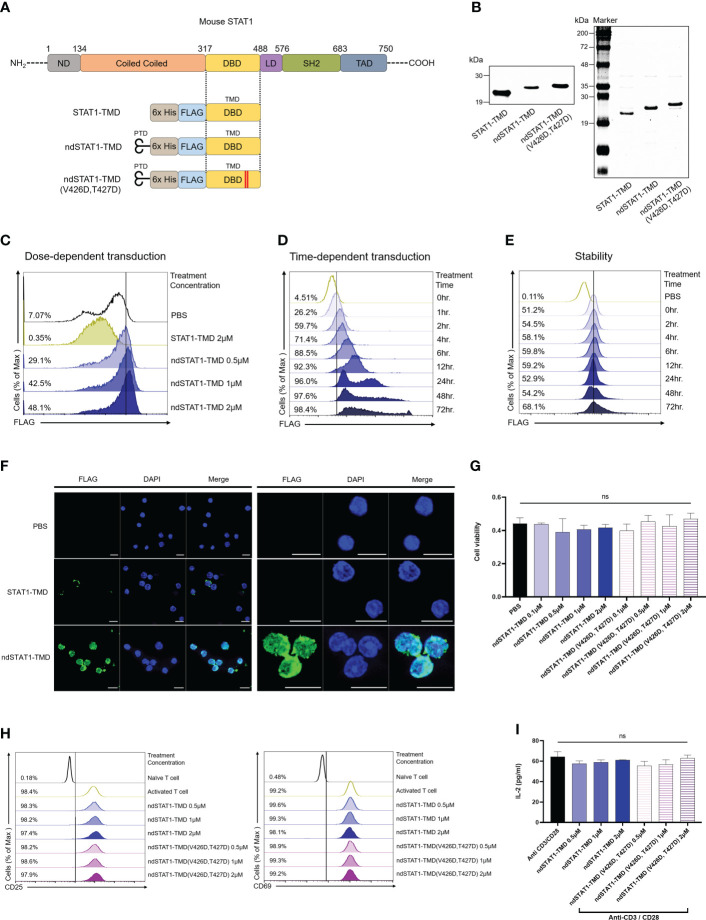
Generation of nucleus-deliverable ndSTAT1-TMD and verification of its intra-nuclear transduction kinetics without cellular cytotoxicity. **(A)** Protein structure of STAT1-TMD without Hph-1-PTD, ndSTAT-TMD, and ndSTAT1-TMD (V426D, T427D) with mutated valine 426, and threonine 427. PTD, protein transduction domain; nd, nucleus-transducible; TMD, transcription modulation domain; DBD, DNA binding domain; ND, N-terminal domain; LD, linker domain; SH2, SCR2 homology domain; TAD, transactivation domain. **(B)** The identity of the purified STAT1-TMD, ndSTAT1-TMD, and ndSTAT1-TMD (V426D, T427D) was confirmed by western blot (left panel) or SDS-PAGE (right panel). **(C, D)** Dose-dependent and time-dependent intra-nuclear delivery of ndSTAT1-TMD (0.5-2 μM) or STAT1-TMD (2 μM) into mouse splenocytes. **(E)** Intracellular stability of ndSTAT1-TMD after transduction into mouse splenocytes. **(F)** Intracellular localization of ndSTAT1-TMD in cultured mouse splenocytes. Representative immunofluorescence 63X (left panel) and 189X (right panel) images using a confocal microscope. Scale bar = 10 μm. **(G)** Evaluation of cellular cytotoxicity of ndSTAT1-TMD or ndSTAT1-TMD (V426D, T427D) in mouse splenocytes analyzed by CCK-8 assay. **(H, I)** The level of the induced expression of CD25, CD69, or IL-2 secretion in CD4^+^ T cells activated by anti-CD3ϵ antibody and anti-CD28 antibody in the presence of ndSTAT1-TMD or ndSTAT1-TMD (V426D, T427D) was analyzed by flow cytometry and ELISA. The graphs are represented as mean ± SEM (n=3), and the statistical analysis was examined using Student’s t-test. ns, not significant.

CD4^+^ naïve T cells can differentiate into Th1 cells by adopting the STAT1 signaling pathway, including the subsequent process of phosphorylation (p-STAT1), homodimerization, and translocation of STAT1 (10). Various genetic and therapeutic approaches have been used to analyze the STAT1 functions *in vitro* and *in vivo*. However, the regulatory roles of STAT1 in the functional context of the T cell network *in vitro* and *in vivo* are still fragmented. To investigate the roles of STAT1 in the functional network of the T cell subset *in vitro* and *in vivo* under normal physiological conditions, we generated ndSTAT1-TMD, a fusion protein between Hph-1-PTD and STAT1-TMD derived as shown in **Figure 1A**. STAT1-TMD without Hph1-PTD was made as a negative control, and the mutated form of ndSTAT1-TMD (ndSTAT1-TMD (V426D, T427D)) was generated in which two essential amino acids (V426, T427) important for DNA binding were changed into D426 and D427 (29). All these fusion proteins were expressed in the *Escherichia coli* system and purified under the native conditions using Ni-NTA agarose. The identity of these purified proteins was confirmed by western blot using anti-6xHis and SDS-PAGE (**Figure 1B**). Next, the intra-nuclear transduction kinetics of ndSTAT1-TMD in the primary T cells was examined. Unlike STAT1-TMD without Hph-1-PTD, ndSTAT1-TMD was effectively delivered into the cells in a dose- and time-dependent manner when treated with 2 μM of concentration (**Figures 1C, D**). The delivered ndSTAT1-TMD remained stable inside the cells until 72 hours post-delivery (**Figure 1E**). Furthermore, when the intracellular location of delivered ndSTAT1-TMD within primary T cells was visualized using confocal microscopy, ndSTAT1-TMD was localized in the nucleus of treated cells but not in the cells treated with STAT1-TMD (**Figure 1F**).

To test whether these fusion proteins have any cell cytotoxicity, primary T cells were treated with ndSTAT1-TMD or ndSTAT1-TMD (V426D, T427D) for an hour with various concentrations and the cytotoxic effect was analyzed by CCK-8 analysis. Neither ndSTAT1-TMD nor ndSTAT1-TMD (V426D, T427D) showed significant cytotoxicity (**Figure 1G**). These data suggest that ndSTAT1-TMD can be effectively and reliably delivered into the nucleus of primary T cells without affecting cell viability.

### ndSTAT1-TMD specifically and simultaneously inhibits the differentiation of naïve CD4^+^ T cells into Th1 and Th17 T cell subsets

T cell receptor (TcR) stimulation is the essential element required for naïve CD4^+^ T cells to commit successful differentiation into various T cell subsets. To examine whether ndSTAT1-TMD or ndSTAT1-TMD (V426D, T427D) treatment of T cells influences the TcR-mediated early activation signaling events, the level of the induced surface expression of CD69, CD25, and IL-2 secretion was measured in the T cells following TcR stimulation in the presence of ndSTAT1-TMD or ndSTAT1-TMD (V426D, T427D). As shown in **Figures 1H, I**, the expression levels CD25, CD69, and interleukin-2 (IL-2) were not affected by ndSTAT1-TMD or ndSTAT1-TMD (V426D, V427D) treatment.

**Figure 2:**
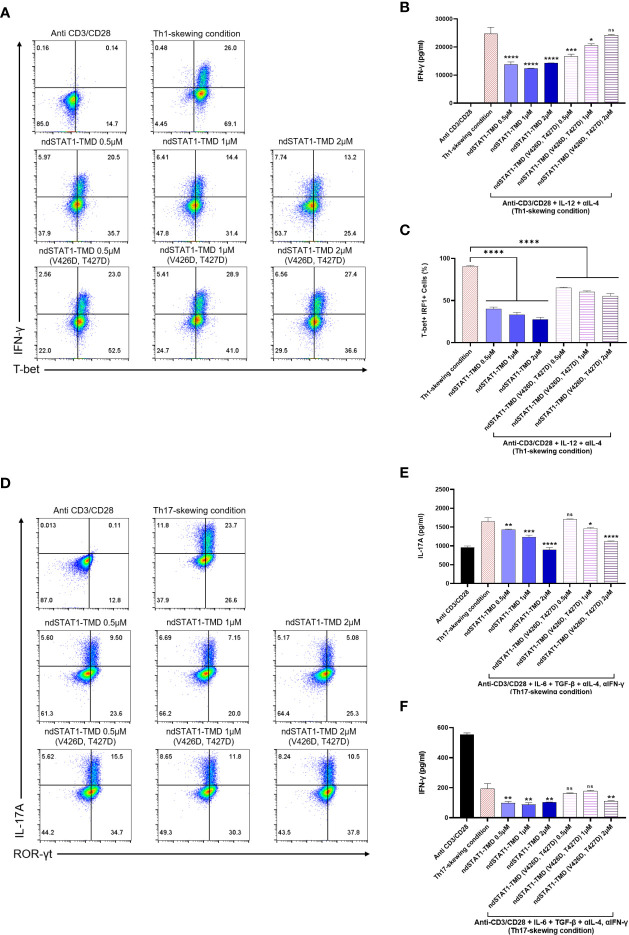
The functional influence of ndSTAT1-TMD on differentiation capacity of CD4^+^ naïve T cells into various T cell subsets. **(A)** Flow cytometry analysis of intracellular T-bet and IFN-γ in Th1 cells treated with different concentrations (0.5-2 μM) of ndSTAT1-TMD or ndSTAT1-TMD (V426D, T427D) under Th1-skewing condition. **(B)** The level of the secreted IFN-γ in the supernatant from Th1 cells in **(A)** was measured by ELISA. **(C)** The level of T-bet^+^ IRF1^+^ cells in Th1 cells in **(A)** was analyzed by flow cytometric analysis. **(D)** Flow cytometry analysis of ROR-γt and intracellular IL-17A in Th17 cells treated with different concentrations (0.5-2 μM) of ndSTAT1-TMD or ndSTAT1-TMD (V426D, T427D) under Th17-skewing condition. **(E, F)** The level of the secreted IL-17A or IFN-γ in the supernatant from the Th17 cells in **(D)** was measured by ELISA. All experiments shown here were repeated 3 times on independent biological samples with similar results. Data are represented as mean ± SEM (n≥3), and the statistical analysis was examined using Student’s t-test. ns, not significant, *p<0.05, **p<0.01, ***p<0.001, and ****p<0.0001.

Next, to investigate the functions of ndSTAT1-TMD during the differentiation of several CD4^+^ T cell subsets, including Th1, Th2, Th17, and regulatory T cells (Treg), naïve CD4^+^ T cells were induced to differentiate into each T cell subset under the appropriate T cell subset-skewing conditions in the presence of different concentrations of ndSTAT1-TMD or ndSTAT1-TMD (V426D, T427D). The level of T-bet expression, intracellular and secreted IFN-γ in Th1 cells substantially decreased by ndSTAT1-TMD treatment in a concentration-dependent manner, and the functional influence of ndSTAT1-TMD (V426D, T427D) on Th1 cell differentiation was significantly less than that of ndSTAT1-TMD (**Figures 2A, B**).

Furthermore, the level of interferon regulatory factor 1 (IRF1), known as a primary responder to IFN-γ-STAT1 signaling in Th1 cells (30), decreased after ndSTAT1-TMD treatment, unlike the Th1 group treated with ndSTAT1-TMD (V426D, T427D) (**Figure 2C**). The expression level of ROR-γt and intracellular IL-17A in Th17 cells was also significantly diminished by ndSTAT1-TMD in a concentration-dependent manner (**Figure 2D** and **Supplementary Figure S1A**). We also found that ndSTAT1-TMD treatment decreased the secretion of IL-17A and IFN-γ from Th17 cells (**Figures 2E, F**). It has been suggested that pathogenic Th17 cells may secrete IL-17A and IFN-γ (31).

Meanwhile, ndSTAT1-TMD showed no influence on regulating the expression of GATA3 or interleukin-4 (IL-4) in Th2 cells and Foxp3 expression in Treg cells (**Supplementary Figures S1B-D**). The ndSTAT1-TMD (V426D, T427D)-treated cells exhibited a recognizable reduction of the expression of the genes associated with the immunological functions of Th1 or Th17 cells. But the level of reduction is substantially less compared to the cells treated with ndSTAT1-TMD at the same concentration. These results suggest that in addition to the binding of STAT1 to the promoter region of the target gene, another functional domain in STAT1 is partially involved in the regulation of Th1 or Th17 cell differentiation (**Supplementary Figures S2A, B**). Taken these results together, our findings demonstrate that ndSTAT1-TMD specifically inhibits the differentiation and functions of both Th1 and Th17 cells without influencing TcR-mediated initial T cell activation signaling events.

### Inhibition of Th1/17 cell differentiation by ndSTAT1-TMD is attributed to the transcriptional suppression of STAT1 target genes associated with the functions of Th1/17 cells

**Figure 3:**
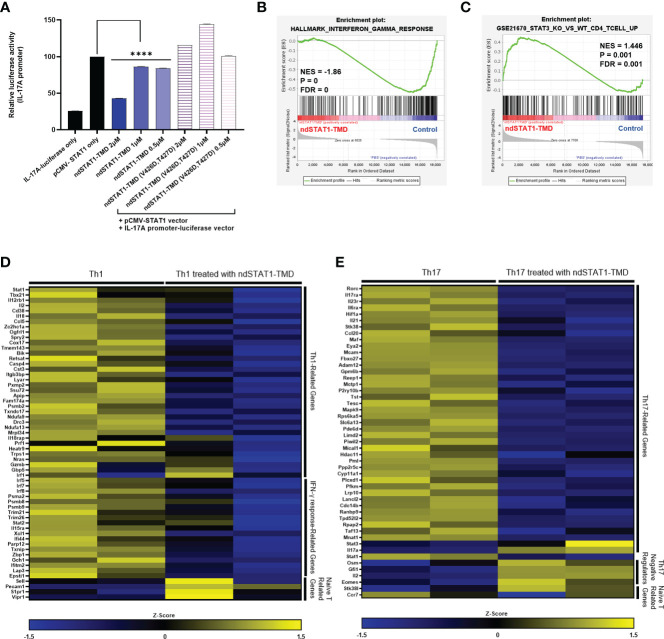
ndSTAT1-TMD inhibits the expression of STAT1-target genes at the transcription level. **(A)** HEK293T cells were co-transfected with the plasmids containing the luciferase reporter gene, whose expression is driven by the IL-17A promoter, and the wild-type STAT1 gene. The luciferase activity of cells was measured 24 hr after the treatment with ndSTAT1-TMD or ndSTAT1-TMD (V426D, T427D) by a luminometer. Data are represented as mean ± SEM (n=5), and statistical significance was indicated when the luciferase activity was lower than the positive control group (pCMV-STAT1-only). The statistical analysis was examined using Student’s t-test. ****p<0.0001. **(B, C)** GSEA-based enrichment plots of the hallmark of IFN-γ response gene set in ndSTAT1-TMD-treated Th1 cells or of the gene set upregulated in STAT3 knockout CD4^+^ T cells compared to wild-type CD4^+^ T cells in ndSTAT1-TMD-treated Th17 cells. **(D, E)** Heatmap of Th1 or Th17 cell signature genes and naïve T cell-specific genes in Th1 or Th17 cells treated with or without ndSTAT1-TMD. Each column means results from one experimental well Z-score was calculated from log2(TPM+1).

Next, we tried to verify whether the suppressive function of ndSTAT1-TMD during the Th1 and Th17 cell differentiation is due to the disruption of endogenous STAT1 binding to the targeted gene promoter at the transcriptional level. Given that STAT1 binds to the IL-17A promoter (32), the plasmid in which the induction of IL-17A promoter drives the expression of the luciferase reporter gene was co-transfected into HEK293T cells along with the plasmid containing the gene encoding wildtype STAT1. Then, the transfected cells were cultured with various concentrations of either ndSTAT1-TMD or ndSTAT1-TMD (V426D, T427D). In the group treated with ndSTAT1-TMD, the luciferase activity was significantly reduced in a concentration-dependent manner. On the other hand, the group treated with ndSTAT1-TMD (V426D, T427D) did not show an inhibitory function (**Figure 3A**). Accordingly, these results infer that ndSTAT1-TMD specifically affects STAT1-mediated transcription within the target cells.

Activated STAT1 is engaged in the generation of IFN-γ-secreting Th1 cells and IL-17A-secreting Th17 cells during the early stage of lineage commitment. Disruption of such functional commitment of Th1 or/and Th17 cells during the differentiation process often leads to developing autoimmune diseases and immune dysfunctions (33, 34). The intrinsic roles of ndSTAT1-TMD exerted on the expression of the genes related to the functions of Th1, and Th17 cells were assessed by analyzing the mRNA expression profile of Th1 and Th17 cells treated with ndSTAT1-TMD. The gene set enrichment analysis (GSEA) demonstrated that the ndSTAT1-TMD-treated Th1 cells showed significant downregulation in the signature genes involved in the IFN-γ response (**Figure 3B**), and the ndSTAT1-TMD-treated Th17 cells followed the gene enrichment pattern of STAT3-knockout CD4^+^ T cells (**Figure 3C**).

Consistent with these GSEA, the mRNA sequencing data from ndSTAT1-TMD-treated Th1 cells indicated that the genes involved in Th1 cell differentiation and functions, such as *Stat1* and *Tbx21*, and the genes necessary for the IFN-γ response, such as *Irf5*, and *Irf7*, were comparatively downregulated, (**Figure 3D**). Likewise, mRNA sequencing data generated from ndSTAT1-TMD-treated Th17 cells demonstrated that the expression of the genes involved in the functions of Th17 cells, such as *Rorc*, *Il17ra*, and *Ccl20*, was significantly reduced, whereas the expression of the genes known to restrict the activation of Th17 cells such as *Osm* and *Eomes* increased (**Figure 3E**). Also, the *Stat1* expression in Th17 cells was reduced, as observed in the Th1 cells (**Figure 3E**). In addition, we found out that the genes associated with naïve T cells were more enriched in ndSTAT1-TMD-treated Th1 and Th17 populations compared to the untreated group (**Figures 3D, E**). Taken these results together, the suppression of Th1 and Th17 cell differentiation by ndSTAT1-TMD is attributed to the competitive binding to the promoter region of STAT1 target genes, resulting in the downregulation of the expression of the genes related to the functions of Th1 and Th17 cells.

### The ndSTAT1-TMD treatment alleviates the symptoms of psoriasis induced by IMQ

**Figure 4:**
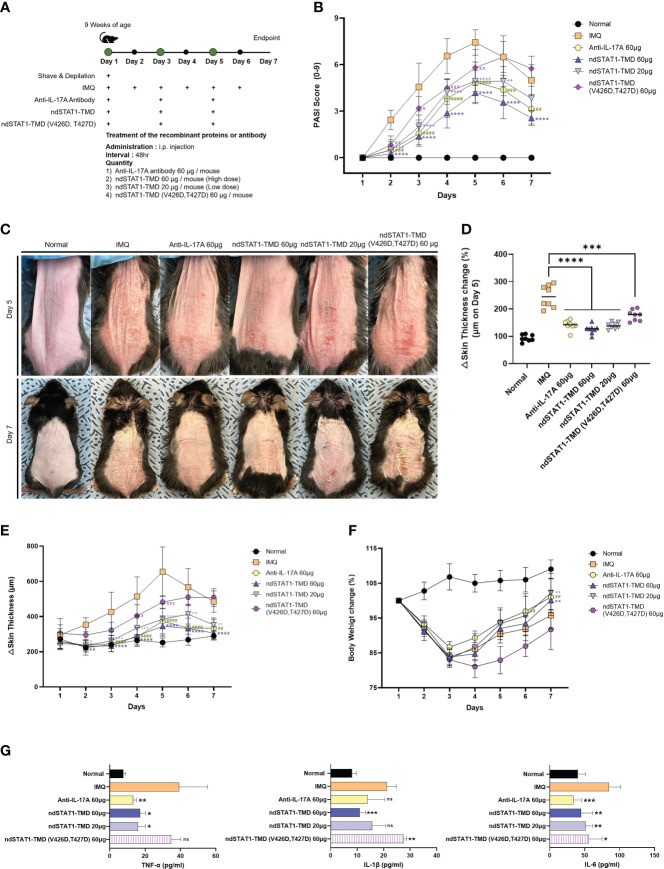
ndSTAT1-TMD treatment attenuates the disease severity of the IMQ-induces psoriasis. **(A)** The treatment scheme of 5% IMQ-induced psoriasis mice with PBS (IMQ group), anti-IL-17A antibody (60 μg/mouse), ndSTAT1-TMD (20 μg/mouse or 60 μg/mouse), or ndSTAT1-TMD (V426D, T427D) (60 μg/mouse). **(B)** PASI scoring plots in the back skin of each group from **(A)** for disease progression. **(C)** Representative images of mice back skin damage from **(A)** on day 5 (above panel) and day 7 (below panel). **(D)** Change of back skin thickness within each group from **(A)** of day 5, calculated based on the thickness of day 1. The graph is represented as mean ± SEM (n=8), and Student’s t-test was used for statistical analysis. ***p<0.001, and ****p<0.0001. **(E)** Back skin thickness of mice in each treated group. **(F)** Body weight change of each group. **(G)** The level of pro-inflammatory cytokines (TNF-α, IL-1β, or IL-6) from the serum of the mice in each group was measured by ELISA. The experiments in the psoriasis model were independently performed twice times. The graphs are represented as mean ± SEM (n=5), and the statistical analysis was examined using Student’s t-test. ns, not significant, *p<0.05, **p<0.01, and ***p<0.001. The graphs in **(B, E, F)** are represented as mean ± SEM (n=8), and the statistical analysis was also examined using ANOVA analysis followed by Dunnett’s multiple comparison test. #,+, x p<0.05; **,##,++,xx p<0.01; ###,+++,xxx p<0.001; ****,####,++++ p<0.0001. * IMQ and ndSTAT1-TMD 60 μg, # IMQ and anti-IL-17A antibody 60 μg, + IMQ and ndSTAT1-TMD 20 μg, x IMQ and ndSTAT1-TMD (V426D, T427D) 60 μg.

Since psoriasis is known as a disease triggered by hyper-activated Th1 and Th17 cells, we next assessed whether ndSTAT1-TMD had therapeutic potential for psoriasis. Psoriasis was induced by applying imiquimod (IMQ) cream to the back and ear skin of mice for 7 consecutive days, and ndSTAT1-TMD, ndSTAT1-TMD (V426D, T427D), or anti-IL-17A antibody as a positive control was administered *via* intra-peritoneal injection every 2 days from the day of initial disease induction (**Figure 4A**) (35, 36). The psoriasis area and severity index (PASI) score measured by analyzing the back skin of the psoriasis-induced mouse peaked on day 5. It then gradually decreased on day 6 or 7 throughout the modeling (**Figure 4B**). The group treated with 60 μg of ndSTAT1-TMD showed the lowest PASI score on day 5 among the treated groups (**Figure 4B**). Likewise, regarding the status of the back skin appearance on days 5 and 7, the ndSTAT1-TMD treatment showed a significant improvement in the pathological changes, comparable to anti-IL-17A antibody treatment (**Figure 4C**). On day 5 from the disease’s onset, the back skin thickness increased 2 folds in the psoriasis-induced group. At the same time, ndSTAT1-TMD or anti-IL-17A antibody treatment substantially reduced the back skin thickness close to the normal group on day 5 and throughout the modeling period (**Figures 4D, E**). The loss of body weight in mice is also considered one of the criteria for measuring disease severity (37). Administration of ndSTAT1-TMD or anti-IL-17A antibody partially prevented the weight loss, consistent with their therapeutic effects (**Figure 4F**). However, ndSTAT1-TMD (V426D, T427) treatment did not show therapeutic activity (**Figures 4B-F**).

We observed that the pathological patterns, such as the PASI score plot and skin thickness change in the ear skin, differed from those in the back skin. Still, ndSTAT1-TMD treatment showed the most effective therapeutic function in the ears (**Supplementary Figures S3A-D**). When the concentration of secreted major pro-inflammatory cytokines such as TNF-α, IL-1β, and IL-6 in the serum of mice in each group was evaluated 7 days after the IMQ induction, the significant decrease of their concentration in the serum was observed in the group treated with 60 μg of ndSTAT1-TMD or anti-IL-17A antibody (**Figure 4G**). These results demonstrate that ndSTAT1-TMD prevents psoriasis progression as effectively as anti-IL-17A antibody.

### The disrupted status of CD4^+^ T cell subsets in secondary lymphoid organs of psoriasis animal model is restored by ndSTAT1-TMD treatment

**Figure 5:**
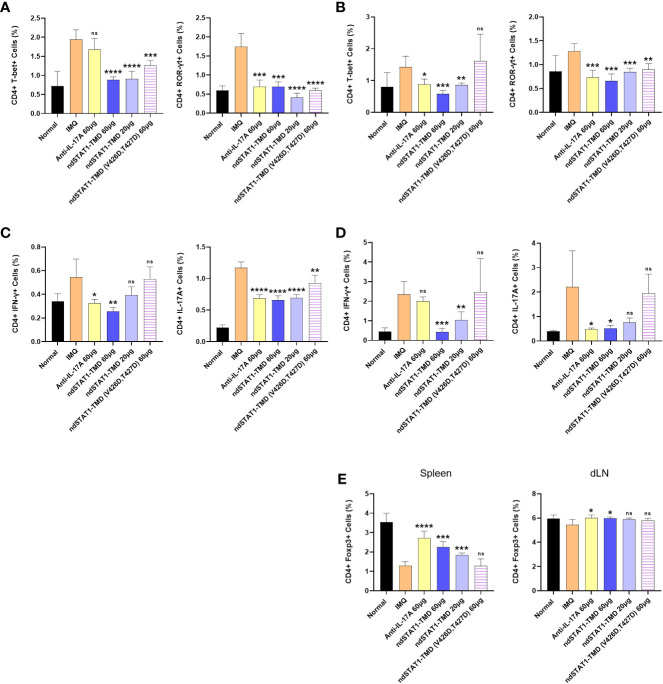
ndSTAT1-TMD treatment restores the disrupted functional status of CD4^+^ T cells in the psoriasis animal model. **(A, B)** The level of CD4^+^ T-bet^+^ T cells (left panel of **(A, B)**) or CD4^+^ ROR-γt^+^ T cells (right panel of **(A, B)**) in the spleen **(A)** and draining lymph nodes (dLN) **(B)** of each treated group was examined on day 7 by flow cytometry. **(C, D)** The level of CD4^+^ IFN-γ^+^ (left panel of **(C, D)**) or CD4^+^ IL-17A^+^ (right panel of **(C, D)**) in the spleen **(C)** and draining lymph nodes (dLN) **(D)** of each treated group was analyzed on day 7. **(E)** The level of CD4^+^ Foxp3^+^ regulatory T cells in the spleen (left panel) or draining lymph nodes (dLN) (right panel) of each treated group. The graphs are represented as mean ± SEM (n=5), and the statistical analysis was examined using Student’s t-test. ns, not significant, *p<0.05, **p<0.01, ***p<0.001, and ****p<0.0001.

Since Th1 cells and Th17 cells are the critical factors for psoriasis pathogenesis and ndSTAT1-TMD inhibited the functions and differentiation program of both Th1 and Th17 cells *in vitro* and *in vivo* (38, 39), we hypothesized that ndSTAT1-TMD treatment of psoriasis animal model changes the functional status of CD4^+^ T cell subsets *in vivo*. When the profile of CD4^+^ T cell subsets in the spleen and draining lymph node (dLN) of the psoriasis animal model was examined, the high level of T-bet^+^ or IFN-γ^+^ CD4^+^ T cells and ROR-γt^+^ or IL-17A^+^ CD4^+^ T cells was detected in psoriasis-induced mice (**Figures 5A-D**). It was observed that ndSTAT1-TMD treatment substantially reduced the level of both T-bet^+^ and ROR-γt^+^ CD4^+^ T cells in the spleen and dLN. Anti-IL-17A antibody treatment showed a therapeutic function relatively specific to ROR-γt^+^ CD4^+^ T cells (**Figures 5A, B** and **Supplementary Figures S4A, B**). Consistent results were also obtained when the level of IFN-γ and IL-17A secretion in secondary lymphoid organs was examined following ndSTAT1-TMD or anti-IL-17A antibody treatment (**Figures 5C, D** and **Supplementary Figures S4C, D**). In contrast to the increase of Th1 and Th17 cells, the level of CD4^+^ Foxp3^+^ T cells was reduced in psoriasis-induced mice. Functional inhibition of Th1 and Th17 cells by ndSTAT1-TMD in the psoriasis-induced mice substantially restored the level of CD4^+^ Foxp3^+^ T cells (**Figure 5E** and **Supplementary Figure S4E**). Collectively, the ndSTAT1-TMD treatment of psoriasis-induced animals showed therapeutic efficacy and restored the functional status of various T cell subsets.

### ndSTAT1-TMD restricts the infiltration of leukocytes into the skin of psoriasis-induced mice

**Figure 6:**
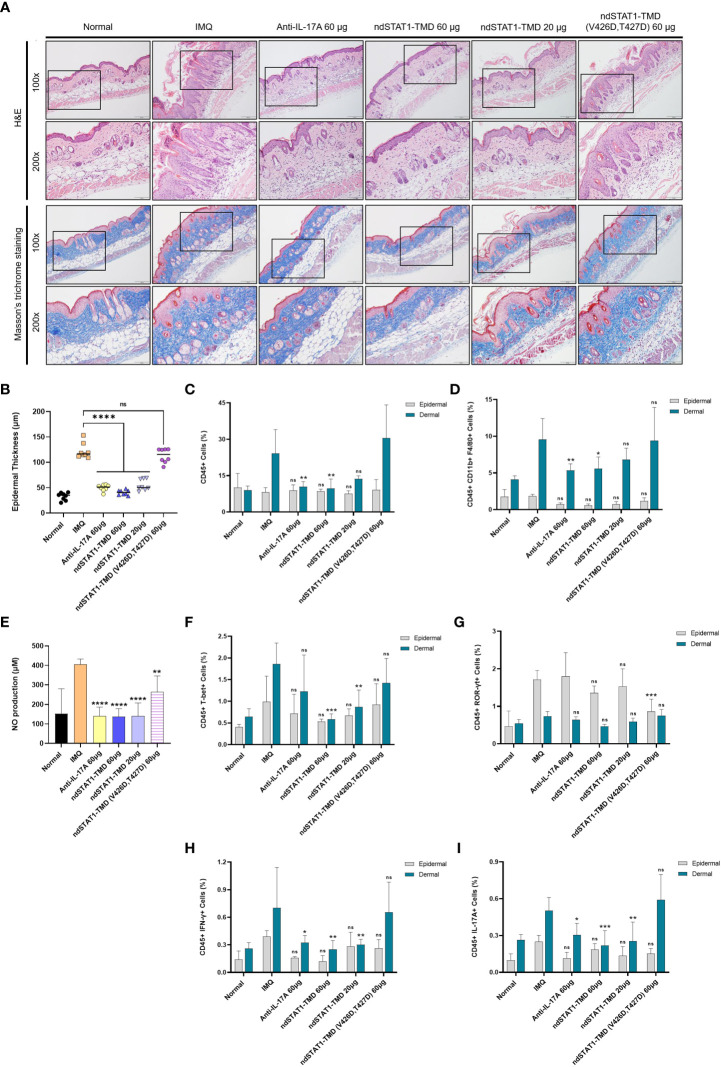
ndSTAT1-TMD treatment of psoriasis animal model prevents the infiltration of leukocytes into the back skin. **(A)** Representative H&E staining (above panel) or Masson’s trichrome staining (below panel) of mice back skin in each group on day 7. Scale bar represents 200 μm for 100X and 100 μm for 200X. **(B)** Change in the epidermal thickness of mice’s back skin of each treated group was measured by the histological images from **(A)**. The graph is represented as mean ± SEM (n=8), and the statistical analysis was examined using Student’s t-test. ns, not significant, and ****p<0.0001. **(C)** The level of infiltrating CD45^+^ leukocytes in the dorsal epidermis (grey) or dermis (blue) area in each group on day 7. **(D)** The level of infiltrating macrophages expressing CD45, CD11b, and F4/80 in the dorsal epidermis (grey) or dermis (blue) area of each group on day 7. **(E)** The nitric oxide (NO) level in the serum of mice in each treated group on day 7. **(F, G)** The level of infiltrating T-bet^+^ **(F)** or ROR-γt^+^ **(G)** T cells in the whole CD45^+^ leukocytes in the dorsal epidermis (grey) or dermis (blue) area of each treated group on day 7. **(H, I)** The level of infiltrating IFN-γ^+^ **(H)** or IL-17A^+^ **(I)** T cells in the whole CD45^+^ leukocytes in the dorsal epidermis (grey) or dermis (blue) area of each treated group on day 7. The graphs in **(C-I)** are represented as mean ± SEM (n=6). The statistical analysis was examined using Student’s t-test, and statistical significance was indicated when the percentage of the cell population was lower than the IMQ group. ns, not significant, *p<0.05, **p<0.01, and ***p<0.001.

It has been demonstrated that the histopathological features of the IMQ-induced psoriasis mouse model closely resemble the multifactorial traits of human plaque-type psoriasis, such as acanthosis, parakeratosis, and neo-angiogenesis, along with the infiltration of various leukocytes consisting of dendritic cells, neutrophils, macrophages, and T cells (40, 41). Several studies have reported that the depletion of CD4^+^ T cells significantly reduces IMQ-induced skin inflammation (40, 42). The H&E-stained sections of the back and ear skin from the psoriasis-induced mice showed the thickening of the epidermis, elongated epidermal rete ridges, hyperkeratosis, and parakeratosis (**Figure 6A** and **Supplementary Figure S5A**). As shown in **Figures 6A, B** and **Supplementary Figures S5A, B**, these psoriasis-associated inflammatory parameters in the back and ear skin significantly decreased after the administration of 60 μg of ndSTAT1-TMD or anti-IL-17A antibody. But the effect of ndSTAT-TMD (V426D, T427D) was minimal. Furthermore, the histological observation of Masson’s trichrome-stained sections of the back and ear skin from the groups treated with either 60 μg of ndSTAT1-TMD or anti-IL-17A antibody showed a significant decrease in the infiltration of keratinocytes and less occupation of collagen fiber in the dermis area (**Figure 6A** and **Supplementary Figure S5A**).

Next, we isolated the leukocytes from the dermis and epidermis of the ear and back skin of the treated mice to delineate the populational change of infiltrating leukocytes. The level of CD45^+^ leukocytes in the dermis of both ear and back skin was diminished in the groups treated with 60 μg of ndSTAT1-TMD or anti-IL-17A antibody, which was not observed in the epidermis area (**Figure 6C** and **Supplementary Figures S5C**, **S6A**). Considering that macrophages located in the dermis area of the psoriatic skin are activated by IFN-γ or/and IL-17A, we examined whether ndSTAT1-TMD can restrict the activation and recruitment of macrophages (43–46). The administration of anti-IL-17A antibody or ndSTAT1-TMD resulted in a significant reduction of CD11b^+^ F4/80^+^ macrophage population in the dermis area of both back and ear skin (**Figure 6D** and **Supplementary Figures S5D**, **S6B**). Consistent with this result, the nitric oxide (NO) level in the serum on day 7 substantially decreased in the groups treated with ndSTAT1-TMD or anti-IL17A antibody, indicating that ndSTAT1-TMD inhibits the activation and recruitment of macrophages (**Figure 6E**).

To investigate whether the reduction of macrophage activation and recruitment to the skin is due to the overall decrease in pathogenic T cell subsets, we analyzed the expression of T cell subset-specific transcription factors and cytokines in leukocytes in the dermis and epidermis area of the ear and back skin. The groups treated with 60 μg or 20 μg of ndSTAT1-TMD showed a significant reduction of CD45^+^ T-bet^+^ T cells in dermal leukocytes (**Figure 6F** and **Supplementary Figures S5E**, **S6C**). On the other hand, the level of CD45^+^ ROR-γt^+^ T cell population in the dermal leukocytes was not significantly affected by the administration of ndSTAT1-TMD or anti-IL-17A antibody (**Figure 6G** and **Supplementary Figures S5F**, **S6D**). Consistently, the group treated with 60 μg of ndSTAT1-TMD showed a substantial reduction of IFN-γ secretion from the dermal leukocytes (**Figure 6H** and **Supplementary Figures S5G**, **S6E**). The IL-17A secretion from the dermal leukocyte was also diminished by treatment of ndSTAT1-TMD or anti-IL-17A antibody (**Figure 6I** and **Supplementary Figures S5H**, **S6F**). Meanwhile, neither ndSTAT1-TMD nor anti-IL-17A antibody treatment exerts any meaningful suppressive effect on macrophages or T cells within epidermal leukocytes of the ear and back skin (**Figures 6D-I** and **Supplementary Figures S5D-H**). Also, ndSTAT1-TMD (V426D, T427D)-treated group did not show an effect on the functional regulation of macrophages and T cells (**Figures 6D-I** and **Supplementary Figures S5D-H**, **S6B-F**). These results demonstrate that ndSTAT1-TMD improves the inflammatory conditions in the dermal area of the psoriatic skin by restricting the activated Th1 and Th17 cells as well as macrophages.

### The severity of DSS-induced colitis is attenuated by ndSTAT1-TMD treatment

**Figure 7:**
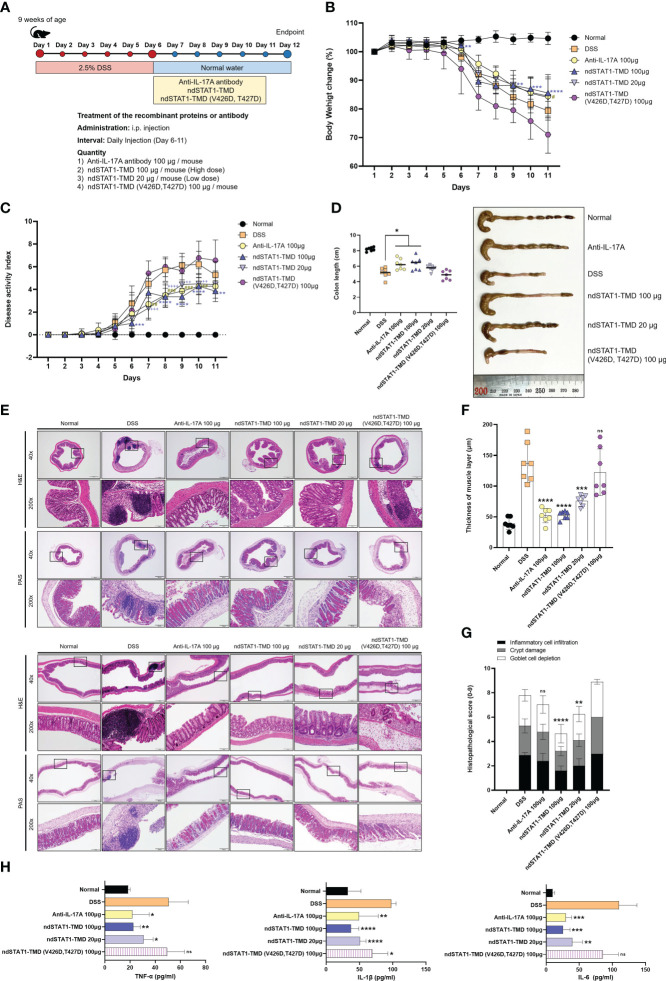
ndSTAT1-TMD alleviates the disease progression of DSS-induced colitis. **(A)** The treatment scheme of 2.5% DSS-induced colitis mice with PBS (DSS group), anti-IL-17A antibody (100 μg/mouse), ndSTAT1-TMD (20 μg/mouse or 100 μg/mouse), or ndSTAT1-TMD (V426D, T427D) (100 μg/mouse). **(B)** Body weight change of each treated group. **(C)** Disease activity index (DAI) score of each treated group. DAI score was calculated by measuring the clinical score of stools, fecal occult blood, and body weight change in each group. #p<0.05; **,##,++p<0.01; ***,###,+++p<0.001; ****,++++p<0.0001. * DSS and ndSTAT1-TMD 100 μg; # DSS and anti-IL-17A antibody 100 μg, + DSS and ndSTAT1-TMD 20 μg. **(D)** Left panel: Comparative analysis of the colon lengths of mice in each treated group on day 12. *p<0.05. Right panel: Representative image of colons from **(A)**. **(E)** Representative vertical (above panel) and horizontal (below panel) colonic sections from **(D)** were stained with H&E or PAS. Scale bar represents 500 μm for 40X and 100 μm for 200X. **(F)** The thickness of the muscle layer of colons in each treated group was measured from **(E)** images. ***p<0.001, and ****p<0.0001. **(G)** Histopathological scores are calculated by inflammatory cell infiltration, crypt damage, and goblet cell depletion. The graph is represented as mean ± SEM (n=6). ns, not significant, **p<0.01, and ****p<0.0001. **(H)** The level of pro-inflammatory cytokines (TNF-α, IL-1β, or IL-6) in the serum of each treated group was measured by ELISA. The graphs are represented as mean ± SEM (n=5). ns, not significant, *p<0.05, **p<0.01, ***p<0.001, and ****p<0.0001. The experiments in the IBD model were independently performed twice times. The graphs in **(B-D, F)** are represented as mean ± SEM (n=7). Statistical analysis was examined using Student’s t-test or ANOVA analysis followed by Dunnett’s multiple comparison test, and in the case of graph **(G)**, statistical significance was shown when the histopathological score was lower than that of the DSS group.

Colitis is another inflammatory disease in which Th1 or/and Th17 cells play pathogenic roles. To evaluate the therapeutic potential of ndSTAT1-TMD in the colitis animal model, a DSS-induced colitis mouse model was generated, and the animals were treated with ndSTAT1-TMD. Anti-IL-17A antibody treatment was used as a positive control (**Figure 7A**). The severity of IBD peaked at 6 days after colitis induction, ndSTAT1-TMD, ndSTAT1-TMD (V426D, T427D), or anti-IL-17A antibody treatment was initiated on day 6 through intraperitoneal injection. And the disease progression was monitored daily for another 6 days.

The rate of body weight loss was significantly improved in the group treated with ndSTAT1-TMD or anti-IL-17A antibody, while ndSTAT1-TMD (V426D, T427D)-treated group continuously lost body weight (**Figure 7B**). The overall disease activity index (DAI) score of colitis substantially decreased in the ndSTAT1-TMD-treated group, comparable to that of the anti-IL-17A antibody-treated group (**Figure 7C**). Consistent with these data, the colon length was maintained well in the group treated with every 100 μg of ndSTAT1-TMD or anti-IL-17A antibody. But the ndSTAT1-TMD (V426D, T427D)-treated group did not show improvement in disease progression or colon length (**Figures 7C, D**). To examine the histopathological status of the colon, we stained the colon sections with H&E or PAS 6 days after the intraperitoneal administration. Indications of the inflamed colon, such as the augmentation of infiltrating inflammatory cells, goblet cell depletion, crypt damage, and the change of muscle layer thickness, were improved in the groups treated with anti-IL-17A antibody or ndSTAT1-TMD (**Figures 7E-G**). On the other hand, the degree of colitis severity remained severe in the group treated with ndSTAT1-TMD (V426D, T427D) without any improvement (**Figures 7E-G**).

The level of secreted pro-inflammatory cytokines such as TNF-α, IL-1β, and IL-6 in the serum from each treated group was examined on day 12. The secretion of these pro-inflammatory cytokines significantly decreased in the group treated with ndSTAT1-TMD in a dose-dependent manner (**Figure 7H**).

In line with the results from the psoriasis animal model, we hypothesized that ndSTAT1-TMD could improve the symptoms of colitis by inhibiting pathogenic functions of the inflammatory Th1 and Th17 cells. In the IBD-induced mice treated with PBS, the level of T-bet^+^ or IFN-γ^+^ CD4^+^ T cells and ROR-γt^+^ or IL-17A^+^ CD4^+^ T cells in the spleen and mesenteric lymph node (mLN) were substantially higher than those in the healthy control (**Supplementary Figures S7A-D**). ndSTAT1-TMD or anti-IL-17A antibody treatment significantly reduced the number of T-bet^+^ or IFN-γ^+^ CD4^+^ T cells and ROR-γt^+^ or IL-17A^+^ CD4^+^ T cells in both the spleen and mLN (**Supplementary Figures S7A-D**). Consistent with the therapeutic potential of ndSTAT1-TMD in the psoriasis animal model, ndSTAT1-TMD showed dual inhibitory functions to Th1 cells and Th17 cells. The decrease of Th1 and Th17 cells by ndSTAT1-TMD treatment was accompanied by the increased number of Foxp3 expressing CD4^+^ Treg cells in the spleen and mLN (**Supplementary Figure S7E**). Taken these results together, the ndSTAT1-TMD treatment also showed therapeutic efficacy in DSS-induced colitis comparable to anti-IL-17A antibody treatment.

## Discussion

It has been well established that immune dysfunction caused by excessive activation of pathogenic CD4^+^ T cells leads to distinctive organ-specific inflammatory diseases. Especially the over- or prolonged activation of Th1/Th17 cells plays a critical role in the onset and maintenance of various autoimmune disorders such as multiple sclerosis, psoriasis, and IBD (47–49). In the case of asthma or atopic dermatitis, overactivated Th2 cells are known to be the leading pathogenic player (50).

STAT1 is an essential transcription factor involved in the differentiation and immunological functions of Th1 and Th17 cells (7, 8). Earlier studies have suggested that genetic deletion of STAT1 is seemingly effective in restricting the hyper-activation of Th1 cells (13, 51). However, the reduced activity of Th1 cells came at the expense of provoking a more significant pathogenic role of Th17 cells within an inflammatory setting, leaving STAT1 a controversial target for preventing overt autoimmunity (13, 52).

To address this issue about STAT1 functions *in vitro* and *in vivo* under the normal physiological condition without using genetic manipulation, we generated nucleus-deliverable STAT1-TMD (ndSTAT1-TMD) comprising TMD of STAT1 and human PTD Hph-1 to modulate the functional activity of STAT1 in the nucleus. ndSTAT1-TMD was effectively delivered into the nucleus of primary T cells and remained stable inside the cells up to 72 hours after delivery without showing any cellular cytotoxicity. The nucleus-transduced ndSTAT1-TMD did not affect the early and late signaling events through TcR stimulation, such as induced expression of CD69 and CD25 and secretion of IL-2.

When CD4^+^ naïve T cells were differentiated into various T cell subsets under T cell subset-polarizing conditions in the presence of ndSTAT1-TMD, the transcriptional activity of endogenous STAT1 was significantly blocked, leading to the specific inhibition of Th1 and Th17 cell differentiation without affecting Th2 and Treg cell differentiation. mRNA sequencing analysis of Th1 or Th17 cells treated with ndSTAT1-TMD demonstrated the expression of genes involved in Th1/17 cell-differentiation and functions such as *Tbx21*, *Irf5* in Th1 cells and *Rorc*, *Il17ra*, *Ccl20* in Th17 cells was significantly down-regulated. Moreover, our data demonstrated that the expression of the genes involved in IFN-γ responses was reduced in Th1 cells treated with ndSTAT1-TMD, and the gene enrichment pattern of Th17 cells treated with ndSTAT1-TMD resembled that of STAT3-knockout CD4^+^ T cells. Of note, ndSTAT1-TMD treatment of CD4^+^ naïve T cells preferentially and specifically exerts the inhibitory roles on Th1 and Th17 cells differentiation without interrupting the differentiation program of Th2 or Treg cells.

Dual suppressive roles toward Th1 and Th17 cells by ndSTAT1-TMD *in vivo* were further confirmed in psoriasis and IBD mouse models in which overactivated Th1 and Th17 cells are critical pathogenic factors. In both disease animal models, ndSTAT1-TMD treatment demonstrated better therapeutic efficacy by modulating the activities of Th1 and Th17 cells compared to the anti-IL-17A antibody, which was relatively effective in blocking the function of Th17 cells. Administration of ndSTAT1-TMD to psoriasis- and IBD-induced mice effectively attenuated the clinical symptoms of diseases and delayed disease progression by restoring the disrupted imbalance of CD4^+^ effector T cells in the secondary lymph organs. Also, in psoriasis mice, ndSTAT1-TMD reduced the infiltration of pro-inflammatory T cells and macrophages into the psoriatic skin sites. Some *in vitro* changes in Th1 and Th17 cell functions and differentiation program and *in vivo* therapeutic efficacy were observed in ndSTAT1-TMD (V426D, T427D)-treated group, suggesting that in addition to TMD, there might be other important functional domains in STAT1.

In this study, it was validated that the novel therapeutic strategy using ndSTAT1-TMD alleviates the symptoms of autoimmunity by modulating the transcriptional activity of endogenous ndSTAT1-TMD in normal physiological conditions without genetic methods. ndSTAT1-TMD specifically inhibited the expression of Th1 and Th17 cell-signature genes that are the main determinants of lineage-specific functions and consequently led to the restoration of the immunological balance in the functional network of T cell subsets.

Secukinumab (Anti-IL-17A antibody) and Fontolizumab (anti-IFN-γ antibody) are being tested in rheumatoid arthritis (RA) (NCT00281294) and psoriasis (NCT04711902, NCT04632927). Considering the results from our therapeutic strategy targeting STAT1 in Th1 cells or Th17 cells, ndSTAT1-TMD can be an effective and new therapeutic reagent for treating various autoimmune diseases associated with pathogenic Th1 and Th17 cells.

## Data availability statement

The original works demonstrated in the study are publicly available. These data are uploaded here: https://www.ncbi.nlm.nih.gov/sra/?term=PRJNA875559.

## Ethics statement

The animal study was reviewed and approved by Institutional Animal Care and Use Committee (IACUC) of Yonsei University (South Korea).

## Author contributions

JP designed the study directions and performed the *in vitro* and *in vivo* experiments, data analysis, and manuscript writing. M-JS helped *in vitro* and *in vivo* experiments. C-CH provided critical intellectual input and helped *in vivo* experiments. S-HL helped *in vivo* experiments and offered helpful input for *in vivo* analysis. YK edited the manuscript and helped with *in vivo* experiments. JA helped *in vivo* experiments. S-KL designed the conception of the study, revised the manuscript, and finalized the approval of the manuscript. All authors contributed to the article and approved the submitted version.
