# Corrigendum: Transcriptional inhibition of STAT1 functions in the nucleus alleviates Th1 and Th17 cell-mediated inflammatory diseases

**DOI:** 10.3389/fimmu.2023.1307575

**Published:** 2023-10-27

**Authors:** Jiyoon Park, Min-Ji Son, Chun-Chang Ho, Su-Hyeon Lee, Yuna Kim, Jaekyeung An, Sang-Kyou Lee

**Affiliations:** ^1^ Department of Biotechnology, Yonsei University of Life Science and Biotechnology, Seoul, Republic of Korea; ^2^ Good T Cells, Inc., Seoul, Republic of Korea

**Keywords:** signal transducer and activator of transcription 1 (STAT1), Th1 cells, Th17 cells, psoriasis, inflammatory bowel disease (IBD), transcription modulation, autoimmune disease (AD)

In the published article, there was an error. The units of measurement for the Vaseline and IMQ cream were incorrectly listed as μg instead of mg.

A correction has been made to **Methods and materials**, *Psoriasis induction and scoring of psoriasis area and severity index*, paragraph 1. This sentence previously stated:

“The normal group applied Vaseline on exposed back skin (40 μg) and both ear skin (20 μg/ear) for 7 consecutive days. And the disease-induction groups were applied with 5% IMQ cream (Aldara cream, Dong-A S&T, Inc. Seoul, South Korea) on exposed back skin (40 μg) and both ears skin (20 μg/ear) from day 1 to day 7.”

The corrected sentence appears below:

“The normal group applied Vaseline on exposed back skin (40 mg) and both ear skin (20 mg/ear) for 7 consecutive days. And the disease-induction groups were applied with 5% IMQ cream (Aldara cream, Dong-A S&T, Inc. Seoul, South Korea) on exposed back skin (40 mg) and both ears skin (20 mg/ear) from day 1 to day 7.”

The authors apologize for this error and state that this does not change the scientific conclusions of the article in any way. The original article has been updated.

