# Supplementary material for: Transcriptional inhibition of STAT1 functions in the nucleus alleviates Th1 and Th17 cell-mediated inflammatory diseases
Source: Front Immunol. 2022 Dec 15;13:1054472. doi: 10.3389/fimmu.2022.1054472 (PMC9800178; doi:10.3389/fimmu.2022.1054472)
Supplement: Supplementary file 1 [file DataSheet_1.docx]

**Supplementary Material**

#
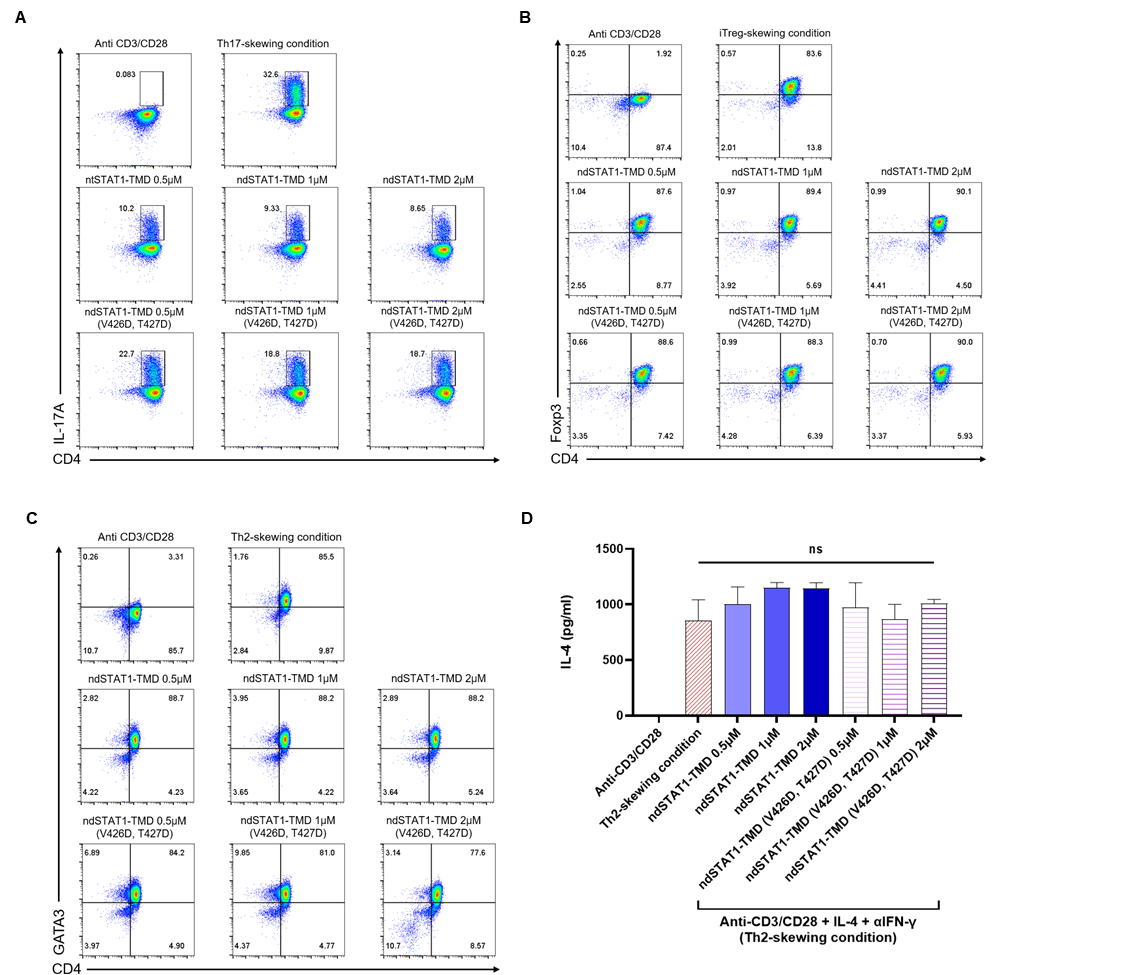
Supplementary Figure 1

**Supplementary Figure 1.** ndSTAT1-TMD does not affect the differentiation potential of naïve CD4^+^ T cells into Th2 and Treg cells. (A) Representative flow cytometry analysis of intracellular IL-17A in CD4^+^ Th17 cells treated with different concentrations (0.5-2 μM) of ndSTAT1-TMD or ndSTAT1-TMD (V426D, T427D) under Th17-skewing condition. (B, C) The effect of ndSTAT1-TMD on Treg cell or Th2 cell differentiation (D) The secreted interleukin-4 (IL-4) concentration in the supernatant from Th2 cells in (C) was measured by ELISA. The experiments were performed at least twice. The graph is represented as mean ± SEM (n≥2), and Student’s t-test was used for statistical analysis. ns; not significant.

#
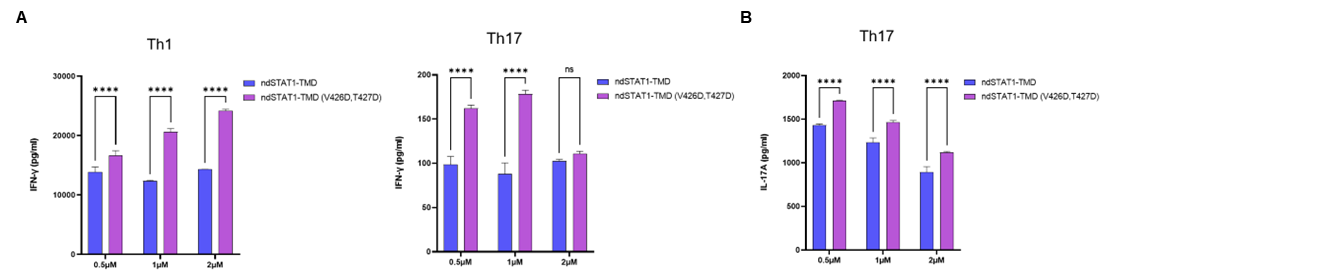
Supplementary Figure 2

**Supplementary Figure 2.** The level cytokines secreted from Th1 or Th17 cells upon the treatment with ndSTAT1-TMD or ndSTAT1-TMD (V426D, T427D). (A) The level of IFN-γ in the supernatant from Th1 cells (left panel), and Th17 cells (right panel) treated with different concentrations (0.5-2 μM) of ndSTAT1-TMD (blue) or ndSTAT1-TMD (V426D, T427D) (purple) were measured by ELISA. (B) The level of IL-17A in the supernatant from Th17 cells treated with different concentrations (0.5-2 μM) of ndSTAT1-TMD (blue) or ndSTAT1-TMD (V426D, T427D) (purple) were measured by ELISA. The graphs are represented as mean ± SEM (n=3) and Student’s t-test was used for statistical analysis. ns; not significant, and ****p<0.0001.


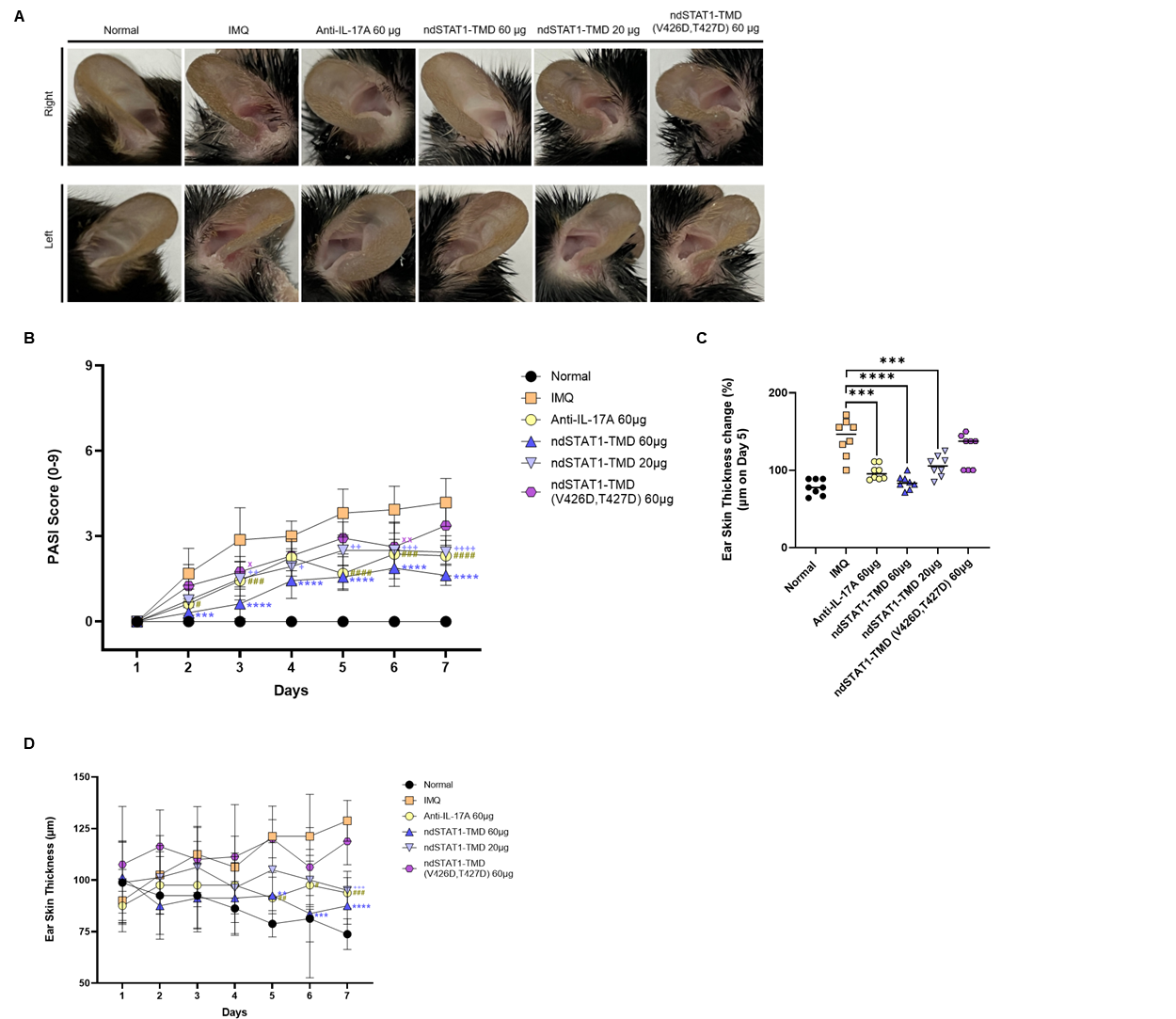
**Supplementary Figure 3**

**Supplementary Figure 3.** Psoriasis severity was measured by skin damage, PASI scores, and thickness in ear skin from IMQ-challenged mice. (A) Representative images of right (above panel) or left (below panel) ear skin damage of mice in each treated group on day 7. (B) PASI scoring plots in the ear skin of each group from (A) for disease progression. (C) Change of ear skin thickness within each treated group from (A) of day 5. The graph is represented as mean ± SEM (n=8), and Student’s t-test or ANOVA analysis followed by Dunnett's multiple comparison test was used for statistical analysis. ***p<0.001, and ****p<0.0001. (D) Ear skin thickness, measured during days 1-7 of mice in each treated group. The experiments in psoriasis model were independently performed twice times. The graphs in (B) and (D) are represented as mean ± SEM (n=8), and the group differences were analyzed by Student’s t-test as statistical analysis. *,#,+,x p<0.05; **,##,++,xx p<0.01; ***,###,+++ p<0.001; ****,####,++++ p<0.0001. * IMQ and ndSTAT1-TMD 60 μg, # IMQ and anti-IL-17A antibody 60 μg, + IMQ and ndSTAT1-TMD 20 μg, x IMQ and ndSTAT1-TMD (V426D, T427D) 60 μg.

**
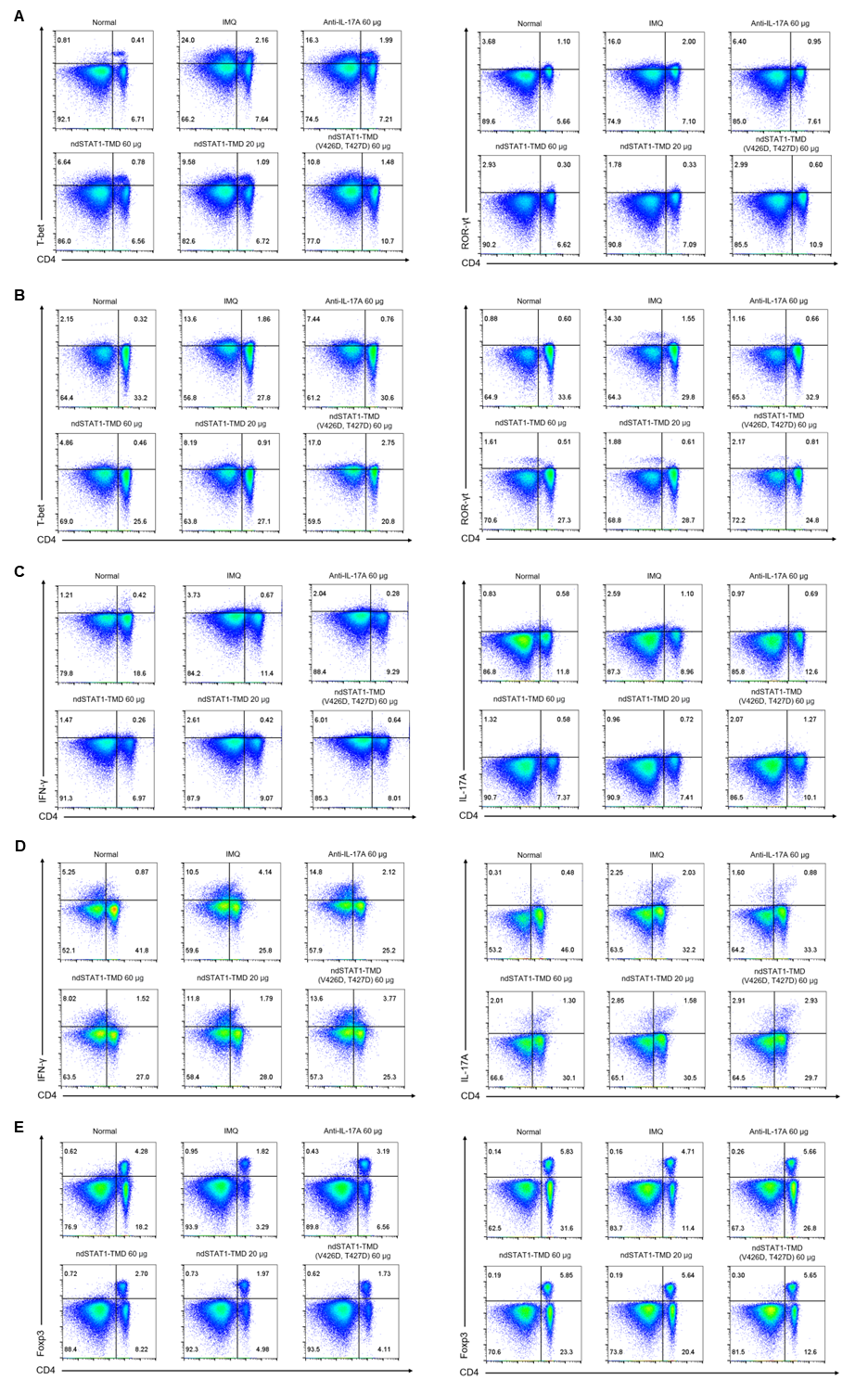
Supplementary Figure 4**

**Supplementary Figure 4.** Flow cytometric analysis for CD4^+^ T cell population in psoriasis mouse model. (A, B) Dot plots representing CD4^+^ T-bet^+^ (left panel of (A) and (B)) or CD4^+^ ROR-γt^+^ T cells (right panel of (A) and (B)) in the spleen (A) and draining lymph nodes (dLN) (B) of each group. (C, D) Dot plots indicating T cell populations expressing CD4 and IFN-γ (left panel of (C) and (D)) or CD4 and IL-17A (right panel of (C) and (D)) among whole lymphocytes in the spleen (C) or dLN (D) of each group. (E) Dot plots representing each treated group's CD4^+^ Foxp3^+^ regulatory T cells in the spleen (left panel) or dLN (right panel). All lymphocytes in the spleen and dLN of each treated group were obtained and analyzed on day 7.

**
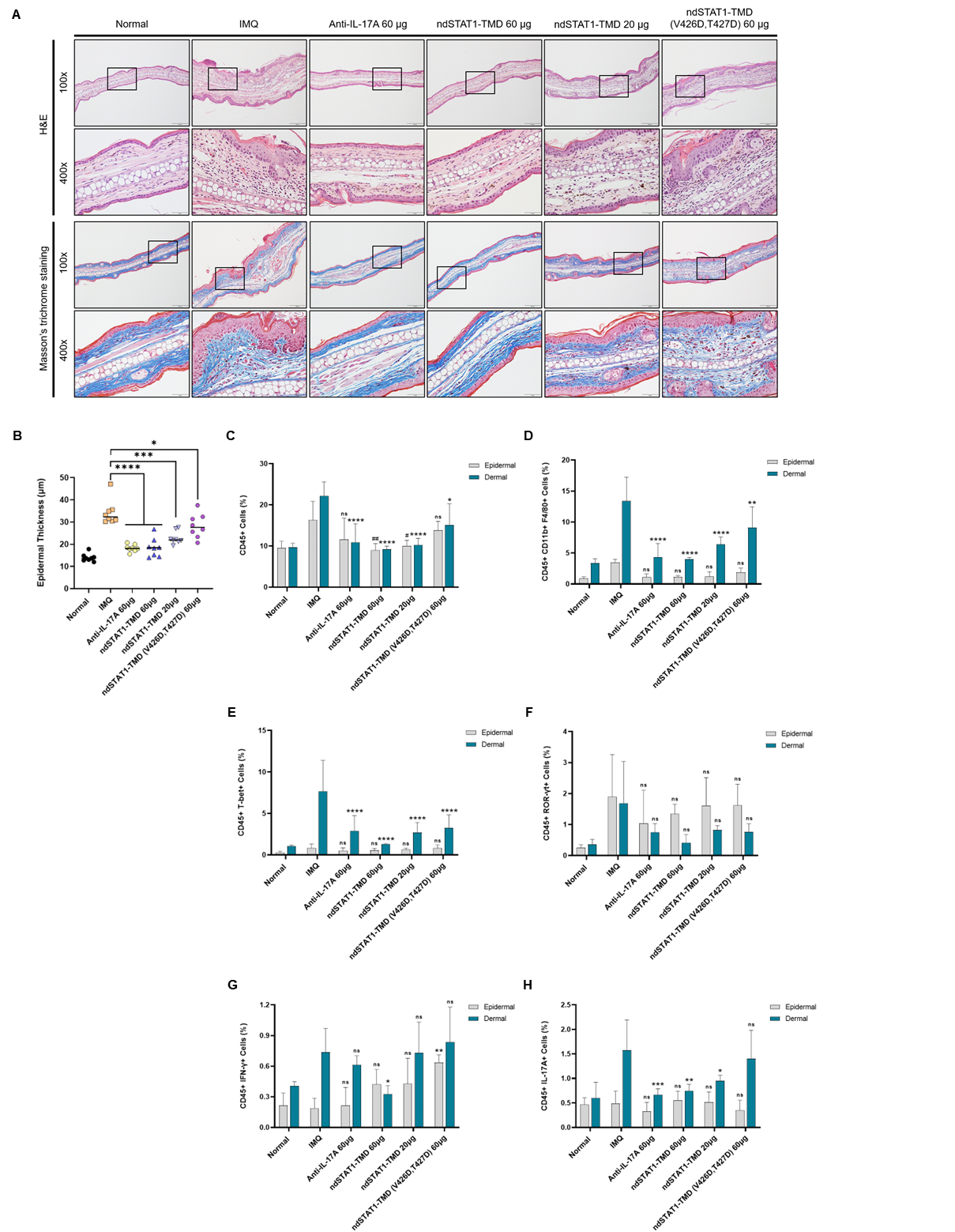
Supplementary Figure 5**

**Supplementary Figure 5.**  Histological analysis and the level of infiltrating leukocyte in the ear skin psoriasis animal. (A) Representative H&E staining (above panel) or Masson’s trichrome staining (below panel) of mice ear skin in each group on day 7. Scale bar represents 200 μm for 100X and 50 μm for 400X. (B) The change of epidermal thickness of mice ear skin, measured by the histological images from (A). The graph is represented as mean ± SEM (n=8). *p<0.05, ***p<0.001, and ****p<0.0001. (C) The level of infiltrating CD45^+^ leukocytes level in the ear epidermis (grey) or dermis (blue) of each treated group on day 7. (D) The level of infiltrating macrophages, expressing CD45, CD11b, and F4/80, in the dorsal epidermis (grey) or dermis (blue) of each group on day 7. (E, F) The level of infiltrating T cells expressing T-bet (E) or ROR-γt (F) among the whole CD45^+^ leukocytes in the ear epidermis (grey) or dermis (blue) area of each treated group on day 7. (G, H) The level of infiltrating T cells expressing IFN-γ (G) or IL-17A (H) among the whole CD45^+^ leukocytes in the ear epidermis (grey) or dermis (blue) area of each treated group on day 7. The graphs in (C-H) are represented as mean ± SEM (n=6). The statistical analysis was examined using Student’s t-test and statistical significance was indicated when the percentage of the cell population was lower than the IMQ group. ns; not significant, *,# p<0.05, ## p<0.01, and ****p<0.0001. * Dermal p-value, # Epidermal p-value.


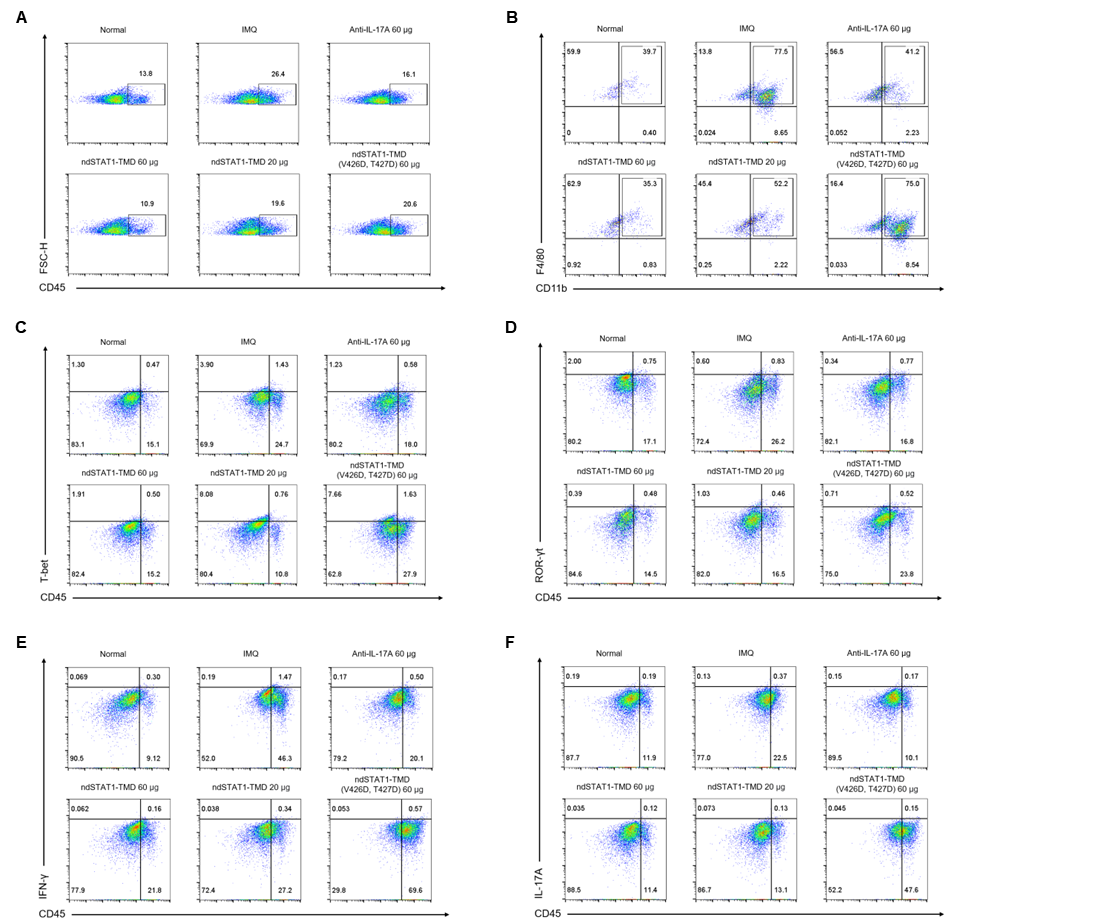
**Supplementary Figure 6**

**Supplementary Figure 6.** Flow cytometric analysis for leukocytes infiltrating into the dermis of back skin in psoriasis mouse model. (A) Representative dot plots representing the infiltrating CD45^+^ leukocytes in the dermis area of the back skin of each group. (B) The population of infiltrating macrophages expressing CD11b and F4/80 among the whole CD45^+^ leukocytes in the dorsal dermis area. (C, D) The infiltrating T cell populations expressing CD45^+^ T-bet^+^ (C) or CD45^+^ ROR-γt^+^ T cells (D) in the dermis area of the back skin of each treated group. (E, F) The infiltrating T cell populations expressing CD45^+^ IFN-γ^+^ (E) or CD45^+^ IL-17A^+^ T cells (F) in the dermis area of the back skin of each treated group. All infiltrating leukocytes into the dermis of the back skin of each treated group were isolated and analyzed on day 7.

**
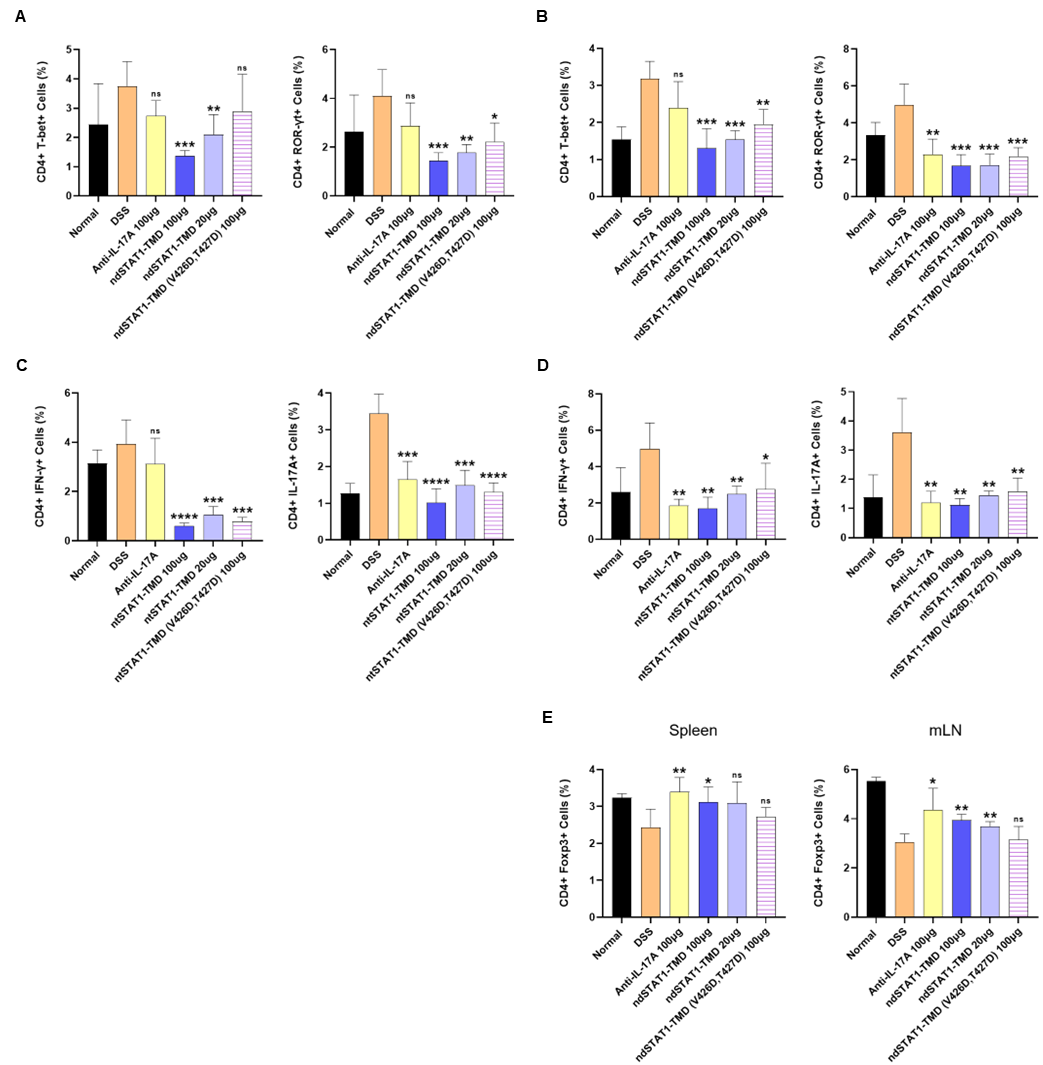
Supplementary Figure 7**

**Supplementary Figure 7.** Analysis for CD4^+^ T cell population in IBD mouse model. (A, B) Percentage of cells expressing T-bet and CD4 (left panel of (A) and (B)) or ROR-γt and CD4 (right panel of (A) and (B)) among whole lymphocytes in the spleen (A) or mesenteric lymph nodes (mLN) (B) analyzed by flow cytometry. (C, D) Percentage of cells expressing IFN-γ and CD4 (left panel of (C) and (D)) or IL-17A and CD4 (right panel of (C) and (D)) among whole lymphocytes in the spleen (C) or mesenteric lymph nodes (mLN) (D) analyzed by flow cytometry. (E) The proportion of CD4^+^ Foxp3^+^ regulatory T cells among whole lymphocytes in the spleen (left panel) or mesenteric lymph nodes (mLN) (right panel) was analyzed by flow cytometry. The graphs are represented as mean ± SEM (n=5), and Student’s t-test was used for statistical analysis. ns; not significant, *p<0.05, **p<0.01, ***p<0.001, and ****p<0.0001.
